# Marginal Probabilistic Modeling of the Delays in the Sensory Data Transmission of Networked Telerobots

**DOI:** 10.3390/s140202305

**Published:** 2014-01-29

**Authors:** Ana Gago-Benítez, Juan-Antonio Fernández-Madrigal, Ana Cruz-Martín

**Affiliations:** Systems Engineering and Automation Department, University of Málaga, Campus Teatinos, Boulevard Luis Pasteur s/n, Málaga 29071, Spain; E-Mails: anagagobenitez@gmail.com (A.G.-B.); jafma@ctima.uma.es (J.-A.F.-M.)

**Keywords:** networked sensors, remote robot operation, stochastic time delays

## Abstract

Networked telerobots are remotely controlled through general purpose networks and components, which are highly heterogeneous and exhibit stochastic response times; however their correct teleoperation requires a timely flow of information from sensors to remote stations. In order to guarantee these time requirements, a good on-line probabilistic estimation of the sensory transmission delays is needed. In many modern applications this estimation must be computationally highly efficient, e.g., when the system includes a web-based client interface. This paper studies marginal probability distributions that, under mild assumptions, can be a good approximation of the real distribution of the delays without using knowledge of their dynamics, are efficient to compute, and need minor modifications on the networked robot. Since sequences of delays exhibit strong non-linearities in these networked applications, to satisfy the *iid* hypothesis required by the marginal approach we apply a change detection method. The results reported here indicate that some parametrical models explain well many more real scenarios when using this change detection method, while some non-parametrical distributions have a very good rate of successful modeling in the case that non-linearity detection is not possible and that we split the total delay into its three basic terms: server, network and client times.

## 1. Introduction

**Figure 1.**
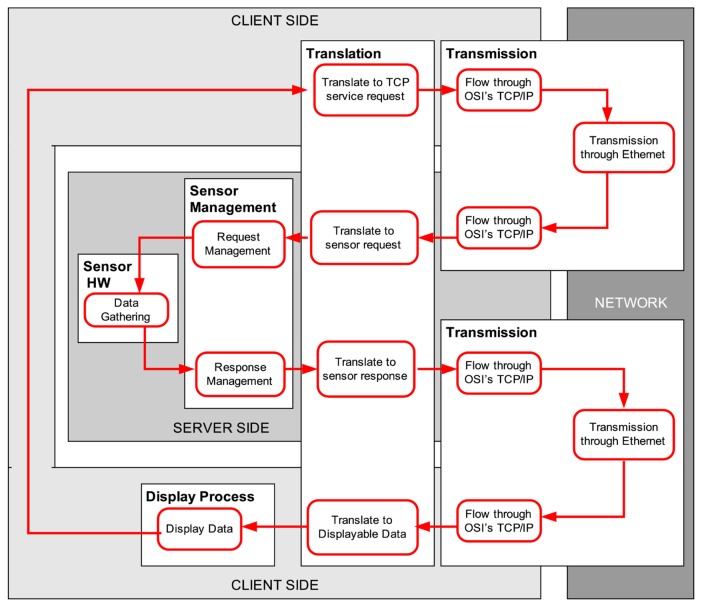
General scheme of a sensory loop in a networked telerobot controlled through the Internet, where both hardware and software components are non-deterministic in the real-time sense.

Telerobots, and, in general, platforms with remotely controlled sensors, are present in many advanced applications, like telecare robotics [1], telesurgery [2], underwater exploration [3], space operation [4], *etc.* In particular, mobile networked telerobots [5] are a class of mobile telerobots controllable over networks like the Internet, that are accessible to the general public through, for example, the World Wide Web. The main operation of such a mobile platform consists of receiving and executing motion commands that are issued from a remote user station, which in turn is displaying information acquired by the sensors of the robot, typically cameras, range sensor data, *etc.* (see Figure 1). This remote control task has time requirements that should be satisfied, because, e.g., the user cannot perform correctly robot navigation if the data are not received from the sensors at the client side with enough frequency.

Current Internet technology presents important problems to perform a hard real-time remote control of these robots due to its stochasticity, but not only the network is an issue in this regard: operating systems and application software, typical of these general-purpose applications, are problematic as well because they may inject unpredictable delays, in some occasions longer than the ones of the network, in a way that makes impossible to guarantee a timely information flow through the control loop in all situations. These are thus *soft* real-time systems [6], where only statistical requirements can be satisfied, for instance “the data from that sensor must arrive every 500 milliseconds with 90% of probability”. A good probabilistic estimation of the delays in the transmission of the sensory data is consequently required to be able to predict the subsequent delays and thus make decisions that guarantee the probabilistic satisfaction of time requirements.

Different approaches, not necessarily robotic, may be found in literature to deal with stochastic delays of this kind: in the networking community they typically try to enhance the Quality of Service (QoS) of the network based on the modeling and control of the information flow [7,8], which usually is addressed by modifications in the network protocols or the hardware [9], or through more exotic approaches such as the injection of artificial delays in the transmission path in order to compensate the already existing ones [10]; in the automatic control area, different theoretical models have been proposed to cope with the full dynamics of remote or distributed systems dedicated to process control—which includes the dynamics of time delays—[11–13], and an important amount of research also is being developed on stochastic networked control systems (NCS), although it is common in these communities to consider the non-network components as deterministic and, usually, to know the dynamics of the plant to control. Finally, approaches to control the timing of data flow in multimedia applications also exist [8,14,15], but they do not need to cope with the strict time requirements of controlling robots. It is noteworthy that most solutions reported in the literature to the modeling and regulation of the delays occurring in networked systems only deal with the network part: typically the end-to-end delay, which includes only A/D conversion, packetization, network propagation, queuing and buffering [16], or the RTT (round-trip time), that refers to the delay existing between sending a package through the network and receiving its acknowledgment [17].

In this paper we are interested in analysing minimalistic mathematical models for all the time delays (not only network delays) found in the kind of applications described before—note that the networked telerobot applications can be generalized, under the perspective of the modeling and regulation of their delays, to any scenario where remote sensors send data to a station through a stochastic network and other non-deterministic software components. The approach presented here pursues to satisfy the following goals: the application requires the data to arrive before a particular time—at least under probabilistic constraints, as explained above—in order to be useful; minimal modifications can be done to the existing system; no previous knowledge is available about the dynamics of the delays; and only a low computational power is available. Taking into account all these constraints, we present a thorough statistical analysis of the time delays of the sensory flow for networked telerobots. We show that temporal sequences of delays gathered from such a system can be successfully modeled with simple statistical tools based on marginal probability distributions, especially when abrupt changes in the signal are appropriately detected—*i.e.*, quickly and with high sensitivity—since those abrupt changes can be then used for separating the sequence into segments that do not contain such non-linearities (in statistical terms, marginal probability distributions are correct models as long as there is temporal independence between values in the sequence). We analyse in the paper the scenarios where this approach is expected to work, and the general characteristics they show. Our marginal distribution approach has reduced computational complexity with respect to other methods, and maintains an appropriate level of accurateness. In particular, it provides statistical significance to the model, or, in other words, the models obtained can explain the data in a statistical sense.

Standard methods that are commonly applied to characterize this kind of sequences of random values work by representing the entire sequence by a single model that captures as accurately as possible all the dependences existing between the values, instead of separating the sequence into nearly independent segments as we propose here. The two most common approaches found in literature are time series and hidden Markov models (HMM), both with well-known computational costs [18] (they are also very often used off-line). On the one hand, time series come in several flavors, depending on their flexibility: ARMA models are *O(m^3^T)*, where *T* is the length of the series and *m* = *p* + *q* the sum of orders of the model—these orders are to be decided previously with some additional procedure— but they are unable to represent signals with abrupt changes or trends; when the series has trends we can use a more involved ARIMA model, which is *O(T^2^)* [19], but it cannot deal with abrupt changes in the signal; when the signal is to be segmented due to the presence of such abrupt changes, real-time algorithms based on ARMA exist that are *O(m^3^T^2^)* [20]; finally, more complex and specific time series algorithms can be found, but with even worse computational costs [21]. On the other hand, HMM deal naturally with signals that change abruptly, representing them as the output of a stochastic process that varies its (hidden) state probabilistically. Unfortunately, learning the parameters of an HMM usually requires a *T* that is longer than in the ARMA case, *i.e.*, to gather appreciably longer sequences of values; in addition, its complexity is *O(N^2^T^2^)*, where *N* is the number of states considered for the system. That number should be estimated previously with some other procedure.

In contrast to these standard approaches, our marginal distribution method is *O(T)*, since the estimation of the distribution parameters and the change detection algorithm are *O(T)* at most: on the one hand, in the worst case when we use a non-linear optimization algorithm to find the parameters of the distribution—which is avoided if we employ maximum likelihood estimation, as it is the case in many of our models, such as the lognormal [22]— the number of optimization steps can be bounded to a constant in all practical cases; on the other hand, the change detection algorithm that we present in Section 4.1 consists in the application of a mathematical expression, which is simply *O(1)*. Also, although our approach is specifically devised to detect and rule out abrupt signal changes, it produces improvements in the modeling of the signal when the delays have smooth (non abrupt) trend changes, since the procedure partitions the sequence of delays into shorter segments where the trend has a relatively lesser significance.

The first result reported in the paper is a statistical analysis, based on a diverse dataset gathered from very different real scenarios, that shows how most typical situations have delays with negligible autocorrelation as long as abrupt changes are detected, and produce long enough near-stationary regimes in that case, thus enabling the assumption of independence and consequently the application of our marginal model. After that, we show how our marginal approach applies to modeling the total delay in the sensory loop of many real scenarios, not only the network delay. Finally, our study proceeds to establish that some heavy-tailed marginal distributions such as the lognormal, along with our confidence interval method for detecting abrupt signal changes, highly improve the modeling of the delays, without requiring any previous knowledge about the dynamics of the system (elsewhere we have reported more in depth results for other heavy-tailed distributions when used with a different, more computationally involved change detection algorithm [23], that in this paper is used only as a near-optimal bound of the results that can be obtained with our marginal approach). The confidence interval method introduced in this work for detecting non-linearities does not overload the control loop—it is intended to be executed at the client side—and thus it is a promising start for further practical applications that use the proposed models in platforms with low computational capacity. Apart from these results with parametrical distributions, we also report results showing that some non-parametrical models are able to model correctly the delays of most scenarios of interest when they are just split into three basic components, something that is easy to do in many situations. This is practical in those cases where we cannot detect and process abrupt changes in the delay signal.

The document is structured as follows: Section 2 describes our experimental settings and justifies in which cases the marginal distribution is a suitable approximation of the stochastic behaviour of the delays; Section 3 proposes theoretical models of the time delays and evaluates their goodness-of-fit when confronted with the unprocessed experimental data; Section 4 introduces the confidence interval method to detect outliers, bursts and regime changes, and provides the results of fitting and hypothesis testing on the same scenarios, complementing them with the illustration of near-optimal results that would be obtained if the change detection algorithm is allowed to be more computationally involved. Finally, some conclusions and future work are outlined.

## 2. Overview of the Experimental Setting

We have set up a number of scenarios (listed in Table 1) for covering the diversity of combinations of non-deterministic components that can be found in many networked telerobot systems, thus obtaining a very complete idea of the possible behaviours of the delays in the transmission of sensory data. Some of these scenarios have been previously used in other works (e.g., [23]), but only with one particular model of the delays, and using different, more computationally involved change detection methods. In this paper the number of scenarios is much larger and we use them for analysing many models simultaneously; we also split some of them into their fundamental parts, and provide, in general, a both wider and deeper analysis of the problem.

One of the scenarios (#13) does not belong to the scope of the problem discussed in this paper: its stochasticity in the delays is practically null. It has been included here for analysing our algorithms in the limit case that delays are almost deterministic instead of stochastic. In reality it could correspond to a very small microrobot with a 8-bit microcontroller on-board, connected to a external PC that requests sensory data through an USB cable (see description below).

The following are the non-deterministic components that set up our scenarios:

- *Remote sensors*: We have used a mobile service robot called SANCHO [27] with: (i) a USB color camera sensor (webcam) with 48-bit color depth and 640 × 480 maximum resolution (it also captures B&W pictures) and (ii) a SICK LMS-200 laser scanner providing 360, 180 and 90 range data, connected through RS-422. The second robot we have employed in our experiments is the Surveyor microrobot [28], which has a CMOS image sensor with 1.3 megapixels requesting 320 × 240 images (5.6% of the full color resolution). The Surveyor robot sends compressed images (JPEG), which randomly alter the actual density of the transmitted data. In the experiments we have considered only this single fixed resolution in order to carry out our analysis consistently. The third robot in our experiments is the Giraff [26], a robotic assistant for the elderly from which we have used the odometry sensors and the camera. We have also included in the experiments some simulated robots: (i) a simple simulation of the odometry of a differential wheeled robot written in PHP (for being executed by the same web server that is receiving the sensor requests); (ii) a complete robot simulation, also in PHP, that includes cameras (only noisy data is served, but with different resolutions); and (iii) a much more realistic simulation written in C++ that have been used in the last decade for a number of robotic experiments in our lab (it resembles the SANCHO robot, including laser scanners, odometry, reactive navigation, *etc.*). Finally, we have used a non-robotic device for simulating microrobots smaller than the Surveyor: an Arduino board [29] that only serves readings from one of the analog input channels of its microcontroller. This system provides much more deterministic delays than all the others, therefore it is especially difficult to model by our stochastic approach.
- *Servers for the sensory system:* The camera sensor of SANCHO is plugged-in to an Intel Pentium IV@3.2 GHz laptop with 1 GB RAM. The laser sensor is connected to a second on-board laptop (Intel Pentium M@2 GHz with 1 GB RAM). Both laptops are connected to the local network through Ethernet, with only twisted pair segments at 1 Gbps (the robot is stopped), and run MS Windows XP and also a robotic software mini-architecture built on the CORBA middleware [30]. The Surveyor robot uses a proprietary firmware that allows a remote client to request specific commands for getting images and other actions. The camera of the Giraff robot and its motion/odometry system are connected to the onboard mini-PC (Intel core i-3-M@2.10 GHz, 3GB RAM, Windows Embedded Standard), which is connected through WiFi to the router of the lab where these experiments have been carried out.
- *Web servers*: For SANCHO, an Apache web server is located in the same sub-network as the robot. It runs GNU/Linux (Ubuntu), and serves a PHP [31] page that connects through TCP/IP sockets to the robot middleware. The Surveyor, in contrast, includes an onboard *ad-hoc* web server that processes remote requests directly. The Giraff robot does not use a web server since it is not managed through a web-based interface, but an *ad-hoc* one [24]. The PHP simulated robots are run by the same web server that receives the sensor requests (on Linux). The C++ simulated robot run on the same machine as this web server, but within a virtual machine with Windows XP. The Arduino system does not have any web server.
- *Web clients*: The client web browser for SANCHO has been Firefox on Linux, where a piece of Asynchronous JavaScript and XML (AJAX) code [31] continuously requests data from the sensors and measures the time delays. Two different laptops have been tested for running this web client: an Intel Core Duo T7200@2 GHz with 2 GB RAM (running Mandriva Linux) and a Pentium M@1.8 GHz with 1 GB RAM (running Ubuntu Linux). Both can use WiFi 802.11b/g connections to access the network: the former through an ISP provider located in Madrid (at about 600 km from our lab); the latter through a local University provider in the same building as the robot. In addition, we have included in some experiments a 56 kbps narrowband segment by using an old phone modem. For the Surveyor robot, a specific Android client application connects to the robot onboard web server, using the robot control protocol and a WiFi *ad-hoc* (single-hop) connection, and retrieving camera images. The Giraff robot is managed through an *ad-hoc* teleoperation graphical interface written in C++ [24], that is run in an external desktop PC (Intel R CoreTM i5 3330@3 GHz, 8 GB RAM, Windows XP). The PHP simulated robots have a simple web interface that has been run on three different computers (Laptop intel core 2 duo T6400@2 GHz, 2 GB RAM, Windows XP; Desktop PC Intel Core i7-940@2.93 GHz, 12 GB RAM, Linux; Laptop Intel Core 2 Duo P9600@2.53 GHz, 4 GB RAM, Linux) with Firefox and Chrome browsers. The C++ simulated robot has the same web interface but run in a different computer (Laptop Intel core 2 duo T7200@2 GHz, 2 GB RAM, Linux). Finally, the Arduino system has no interface: direct sensor requests are conducted through the USB connection from a testing program, also written in C++, that runs on a Desktop PC Intel core i7 960@3.20 GHz, 12 MB RAM, Linux.

As we have already reported elsewhere [23], since our approach is aimed at not using any knowledge about the plant that is producing the delays, its applicability cannot be demonstrated analytically for every existing combination of network, computer hardware and software, but notice that the components enumerated above are representative of a wide range of practical, non hard real-time applications: concerning the network, some of our setups include a number of hops, since they have been carried out from geographically distant places—including transatlantic submarine Internet cables—while others have been performed just with one network segment in the system or even a simple point to point connection; the hardware of the robots is obviously fixed, but both are very different and in particular have different computing power, based on a microcontrollers, standard laptops and mini-PCs; in addition we have used common robotic sensors, which include cameras to justify the applicability of our approach to a wide diversity of scenarios, because they allow us to largely vary the amount of data transmitted; finally, the software used in all the experiments is for standard general-purpose machines (except for the Surveyor firmware and the Arduino board), and we have no restriction in working with any reasonable number of applications, not related to our experiments, while they were executed, including modules in charge of other tasks in the robot, office software, Internet navigation in the case of the client controller (with different web browsers and OSes), and other applications in the case of the web server.

Moreover, the state-of-the-art literature on networked telerobots shows that these components are the most common in this kind of robots (see, for instance, [32,33]). In [34] you can consult a list of usual telerobots and camera sensors. Regarding the communication links, [32] presents a survey of wired and wireless connections (they refer to 2006, though the technologies presented are still valid). In Table 2 we summarise two examples of this kind of robots and their components.

In stochastic systems like the ones described before, the total delays of the sensory loop (*i.e.*, network + robot + client and server software) may vary over time due to different causes: transmissions from other sensors, network congestion, non-real-time operating systems in the loop, unpredictable behaviour of the client and the robot software applications, *etc.* Note however that it is unlikely that the delay produced when requesting data from a sensor depends on the values of previous delays measured for the same sensor, provided both that the sensor is not requested again until the previous requests end, and that the system has no explicit memory of these delays. Therefore, our hypothesis is that the dependences appearing in a sequence of delays will be strongly determined by changes in the underlying stochastic state of the components of the system, out of our reach.

**Figure 2.**
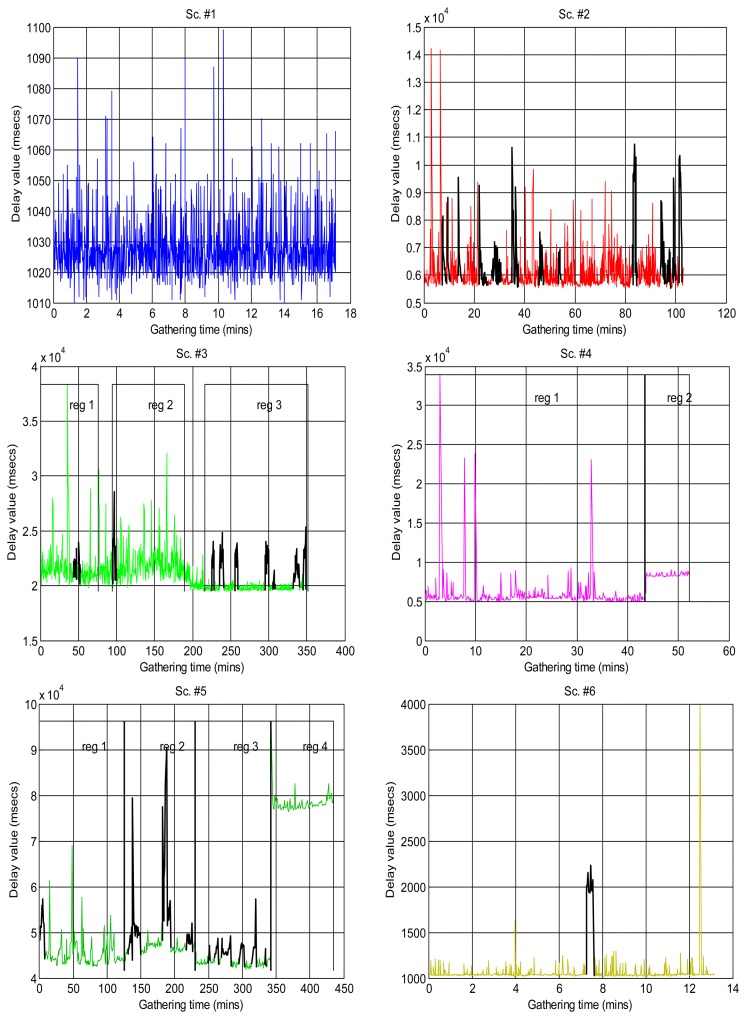

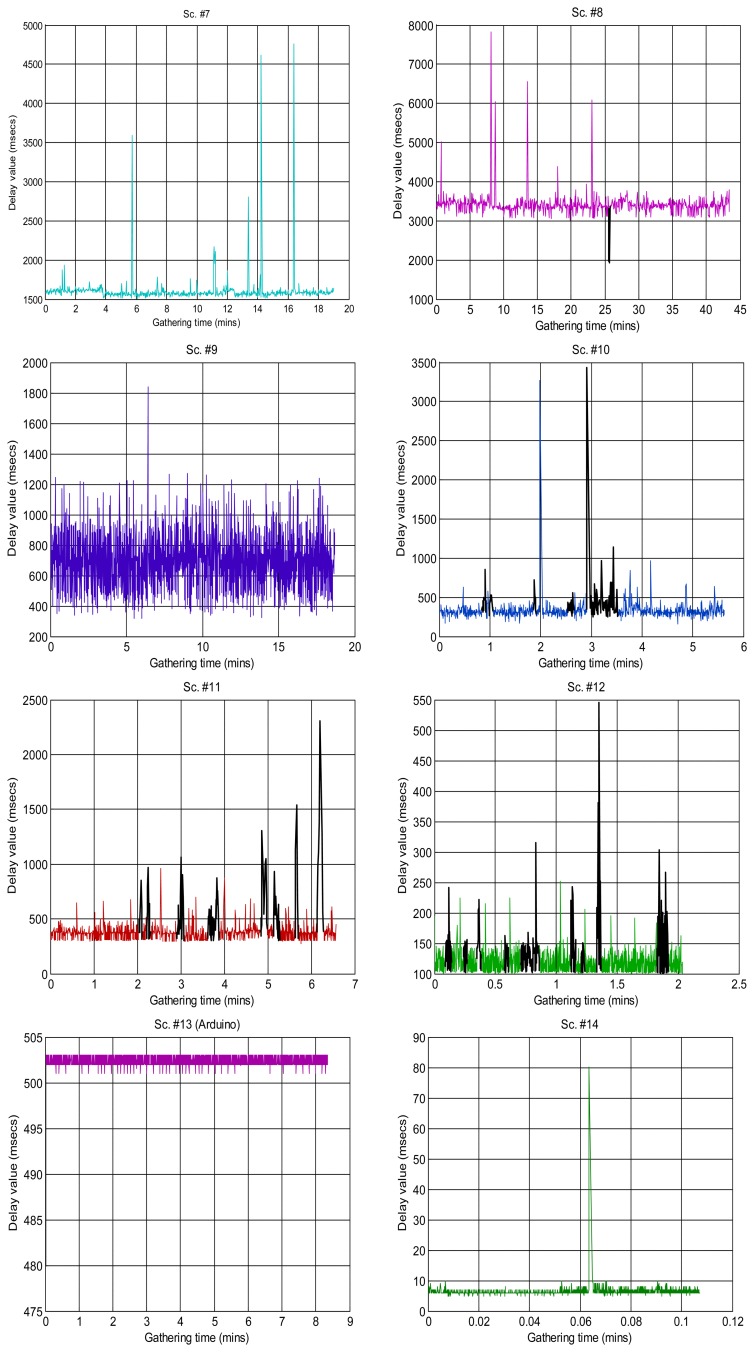

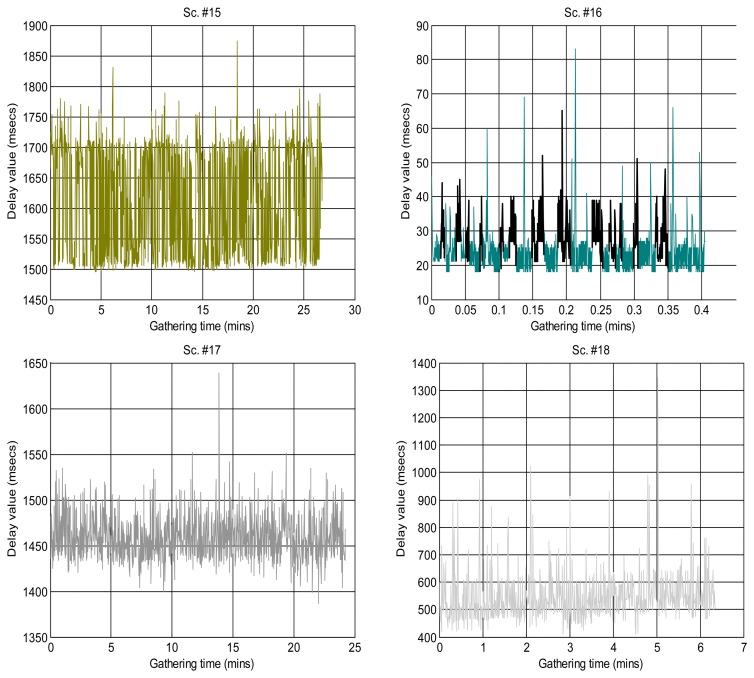
Real delays, in milliseconds, gathered from the sensory loop of the scenarios of Table 1. They present bursts and regime changes (visually detected with black lines), and in some cases smooth trend variations. The bottom x-axes show the absolute time when each sample was gathered. Note the highly deterministic behavior of scenario #13.

**Figure 3.**
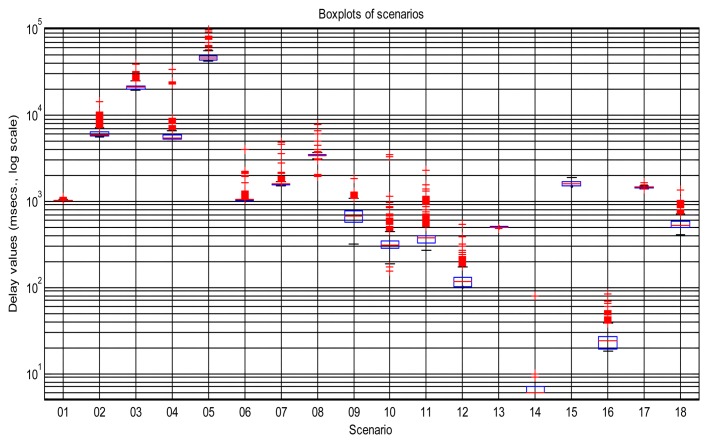
Boxplots of the scenarios of Figure 2. The y-axis is shown in logarithmic scale to appreciate better the amount of delay values (red markers) that are above the principal quartiles (blue boxes), which demonstrates the skewness of the marginal distributions.

Figure 2 shows the sequences of total delays that we have measured in the sensory loop for each of the scenarios defined in Table 1, versus the time when each delay value was measured; it is easy to observe abrupt changes of regimes, outliers and bursts in those delay signals. Also, some of them exhibit smooth changes in their average, *i.e.*, their trends vary. In that figure we have drawn thick lines for separating portions of the scenarios where we cannot detect visually relevant abrupt changes or other non-linearities (*i.e.*, these portions are near-stationary, also called “regimes”), and marked some clear bursts. The boxplots of Figure 3 confirm the wide range of time delays measured and the pronounced skewness of their marginal distributions.

**Figure 4.**
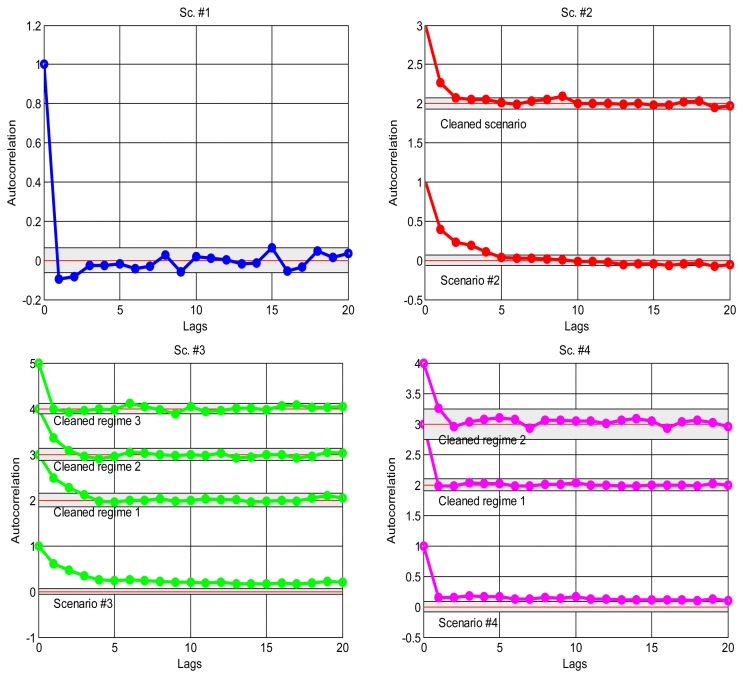

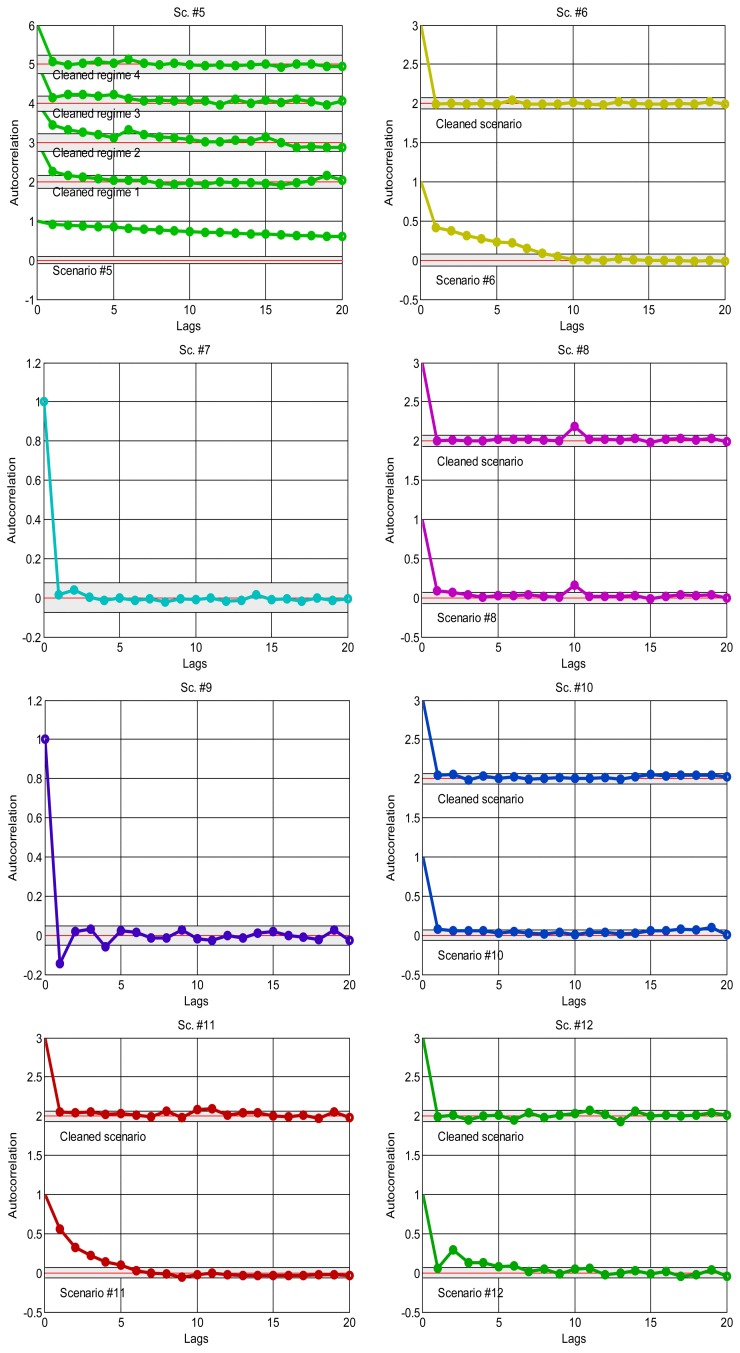

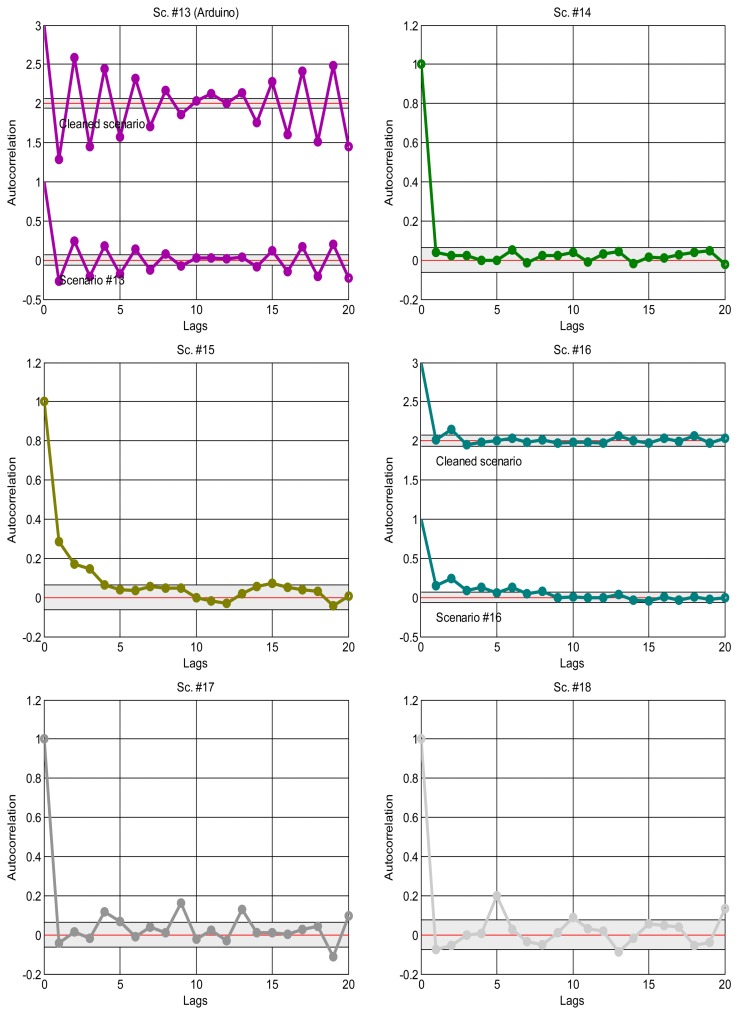
Autocorrelograms of the original scenarios of Figure 2 and of the visually purged scenarios once they are divided into the visual regimes marked there. Observe that in the cases when some lags go above/below the confidence limits, they do not exceed the 5% of the total count of lags (except for scenario #13).

As explained in the introduction, it is a suitable approximation to assume statistical independence of sequential delay values in the stationary parts of the signal, which will allow us to apply the basic tools of the next sections and, in general, work with marginal distributions as suitable models of the data. To confirm this, we have analysed the correlation coefficients in the acquired data: the delays would be dependent if the autocorrelogram function (ACF) goes above the corresponding confidence limits; otherwise, our independence hypothesis cannot be rejected [35]. Figure 4 shows the ACFs of the scenarios, where it is clear the strong dependence of many of them when non-stationarity is not detected and handled, and also the ACF of the visually purged scenarios (eliminating the visually detected bursts and regimes marked in Figure 2). We can see how the ACF stays closer to zero in the latter, and it remains below the confidence limits in most cases. This supports our assumption of considering *iid* sequences of delays (independent and identically distributed) as long as they are separated in regimes and we can also detect bursts and isolated outliers. Of course, smooth variations on the underlying distribution—smooth trend changes—are still possible, but the results obtained with our abrupt detection method also applies in many cases that exhibit such trending.

For this article, we have classified the scenarios of Table 1 into three different categories, depending on the kind and combination of non-linearities that can be observed in Figure 2. This classification is subjective and a priori, but it is interesting because it reflects some important features of the signal. We have defined, by simple visual inspection of the data, three classes:

- Some of the scenarios do not exhibit clear abrupt changes, but do show a smooth fluctuating trend instead. We set these as class A (scenarios #1, #9, #3reg1—corresponding to the left part of scenario #3, Figure 2, which we have visually split into two regimes #3Reg1 and #3Reg2—, #10, #12, #15, #16, #17).
- Another broad group of scenarios do not show any continuous, smooth changes in their trend, and may or not have abrupt changes of regimes or bursts. They are class B, which can be further sub-divided into sub-class B1, on the one hand, for the scenarios that do not have those abrupt changes or that have only one dominant regime in the signal (scenarios #2 and #18), and sub-class B2, on the other hand, for scenarios #3reg2, #4, #5, #6 and #7.
- Finally, we have consider a special class C for scenarios that have neither abrupt changes of regime or bursts nor relevant fluctuation of their trends—thus, in principle they could be in class B—but that present outliers that are much smaller than the majority of its sequence of delays, something that does not occur in other classes and that we believe can have consequences in its modeling with many of the distributions explained in Section 3.1—due to the fact that their histograms do not follow the typical shape of a long-tailed distribution on its left-side portion. Scenarios #8, #11, #13 and #14 can be assigned to this class.

One purpose of this preliminary visual classification is to illustrate better (*i.e.*, more intuitively) further experimental results. In principle, those results should go as follows: class B1 should produce good models due to its lack of abrupt changes and trend variations, and the same should occur for class B2 as long as abrupt changes are detected, since that can be enough to reduce the effects of the trends; class A is in a similar situation as B2: it can produce good models as long as the detection method reduces the mentioned effects of the changing trends; finally, it is not clear how class C will be modeled by our approach, since in principle it should have good models for its lack of abrupt changes and trends, but the presence of the special outlier makes that uncertain.

## 3. Modeling Unprocessed Delays

In this section we present how the original scenarios, without filtering out their non-linearities, fit directly with parametrical, non-parametrical and mixed marginal distribution models, which is assessed by two goodness-of-fit tests. This illustrates the basic methods employed (summarised in Section 3.1) and states their limitations in that initial situation. After showing these modeling results in Section 3.2, Section 3.3 is devoted to improve them—still without filtering the data—through a simple splitting of each delay into three different additive parts that correspond to the three general components that constitute our networked telerobot: the delay in the client side, in the network, and in the server (robot). We analyse whether that splitting, which involves a negligible overhead in the algorithms and in the modifications required on the system, leads to better results.

### 3.1. General Settings

Our main analysis procedure has two steps: (i) a fitting of a certain theoretical marginal model with a large enough set of delays selected from the real scenario; and (ii) a hypothesis test where the rest of the delays of the scenario are used for assessing the suitability of that fitting. We have used even index delay values from each original sample for the fitting step, while odd index values have been selected for the statistical assessment of the goodness of the model (hypothesis testing). This is to preserve as best as possible the same autocorrelations in the temporal sequence of the signal in both steps. Also, using this division we decouple fitting from hypothesis testing, avoiding the reduction in degrees of freedom and allowing us to use tables that exist for some of the statistical tests [36].

For modeling the marginal stochastic behaviour of the sequences of delays we have chosen both parametrical (listed in Table 3) and non-parametrical probability density functions (pdfs). We have not included symmetrical distributions like the normal (proposed in some works, such as [14,37]) because of the pronounced skewness of our scenarios. The models we have selected reflect some insights about the characteristics observed in the real data, namely:

- When the system has very short delays—for example when the sensors send few data, as it is the case with the typical range sensors or the odometry of a mobile robot, or when the communications and/or software run really fast—the shape of the statistical distribution of the delays is very close to an exponential [38]. Actually, exponential distributions are used for modeling inter-arrival times in many situations [39]. In order to make this model more flexible we have also included in our analyses the erlang distribution, which can explain data coming from a sum of *k* independent exponentially distributed components.
- In the networking literature, long-tailed and heavy-tailed distributions are often assumed for the transmission delays in the network when the delays are not so short. For instance, lognormal distributions have been proposed elsewhere [40]. To our knowledge there is no report in the literature showing that the total delay (*i.e.*, network + client + server) supports this model, but our final results corroborate its suitability for more scenarios than other models; furthermore, when the three additive components of the total delay are split (Section 3.3), or when we use our change detection method (Section 4.2), the lognormal becomes the parametrical model of preference.
- In order to increase the feasibility of the previous mentioned models we have also tested the Weibull distribution, which is a generalization of the exponential. For the same reasons we have included the gamma distribution, which generalizes the erlang and has been proposed for modeling the round trip time of wireless network transmissions [17].
- Non-parametrical models allow for greater flexibility in their shapes than parametrical ones, which has pros and cons: they can fit better more complex behaviours, such as the ones in unprocessed scenarios, but they are more likely to model the variations of a particular sample rather than the underlying stochastic process of the whole signal (*i.e.*, overfitting), being poor models when new samples are gathered. We have included two non-parametrical models in this work: one based on a Gaussian kernel [41], able to model signals with more than one mode (which can be useful for sequences of delays that have more than one regime), and a spline distribution consisting of a cubic polynomial sequence that interpolates a set of control points [42]. The latter is more likely to suffer from overfitting, since it imposes the least rigidity on the data.
- Finally, we have also included in our analyses a hybrid model that draws advantages from both parametrical and non-parametrical representations: a sum or mixture of gaussians (SoG), which is less likely to suffer from overfitting than the spline and is more rigid than the kernel. This model consists of a sum of a given, fixed number of Gaussians (this is different from the Gaussian kernel model, which can have an unlimited number of kernels). We have chosen specifically four Gaussian modes for this SoG, which has been deduced visually from the multimodal behaviours of Figure 2. Its parameters have to be estimated with a special trust region non-linear optimization algorithm, which is implemented by the Curve Fitting Tool of Matlab [43].

For finding the best fitting for any of these models, a histogram of the data is constructed first with 2*n*^2/5^ cells, where *n* is the fitting sample size. We use then maximum likelihood estimation (MLE) for each distribution (or the trust algorithm in the case of the SoG). After this fitting, in order to study the goodness-of-fit, we have employed both the Chi-Squared (χ^2^) and the Kolmogorov-Smirnov (K-S) significance tests with 0.05 of significance. As it will be shown further on, the former gives worse results in general, particularly due to its higher sensitivity to shorter samples (something that is likely to occur when detecting abrupt changes and separating the sequence of delays into segments, as it is shown in Section 4.2).

### 3.2. Results for Unprocessed Delays

**Figure 5.**
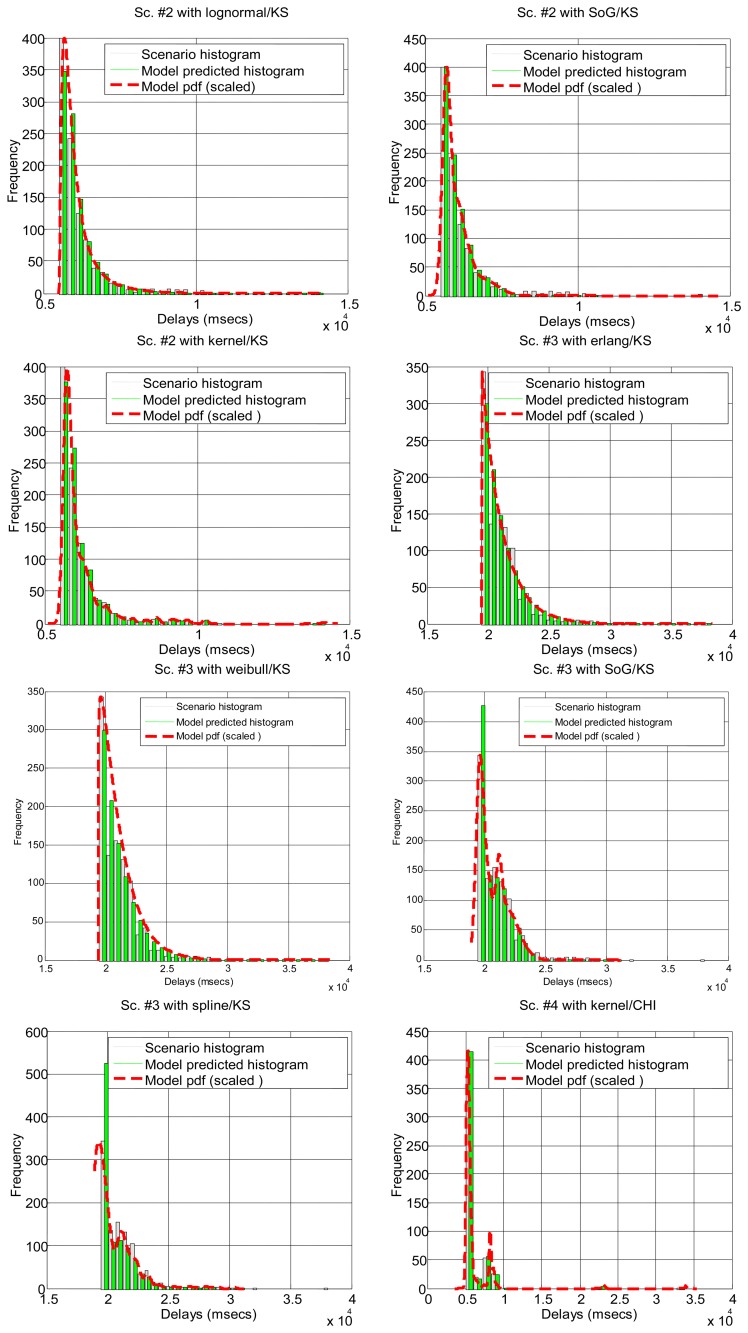

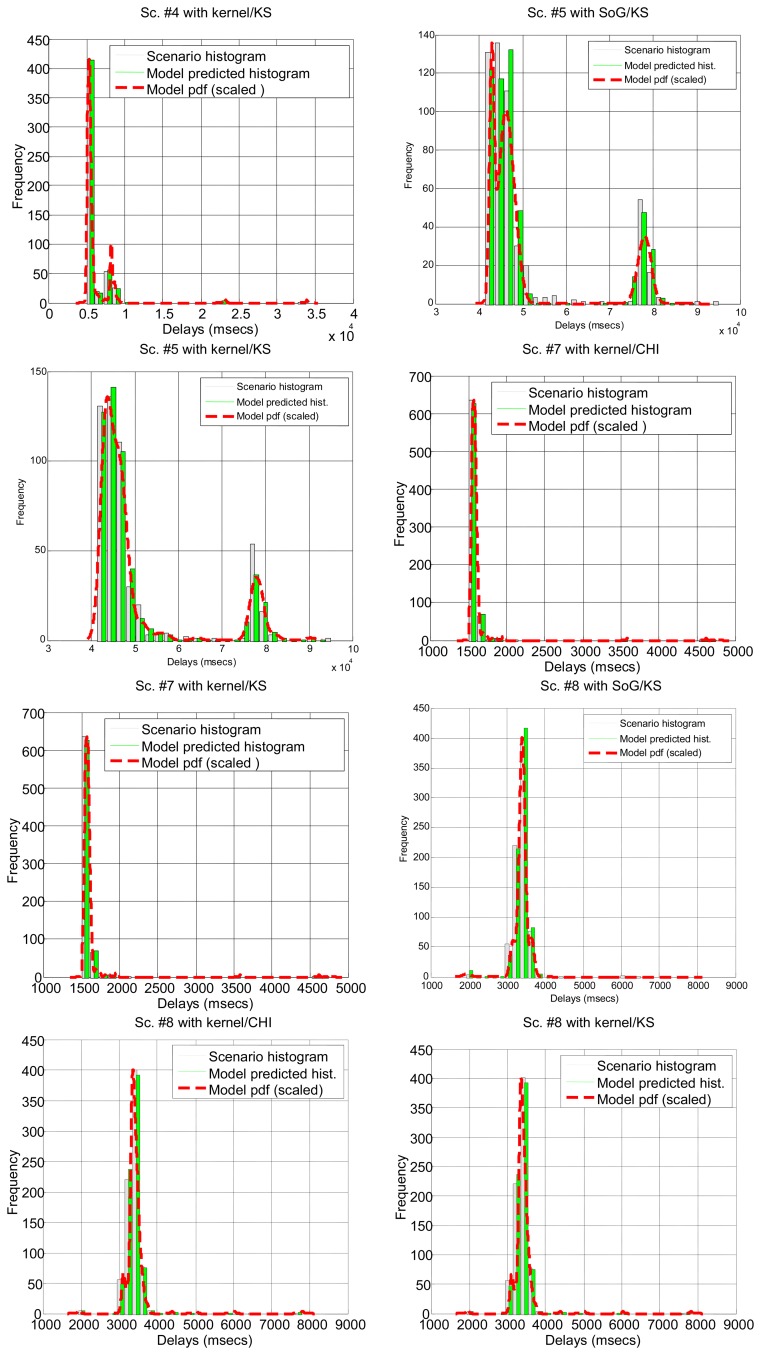

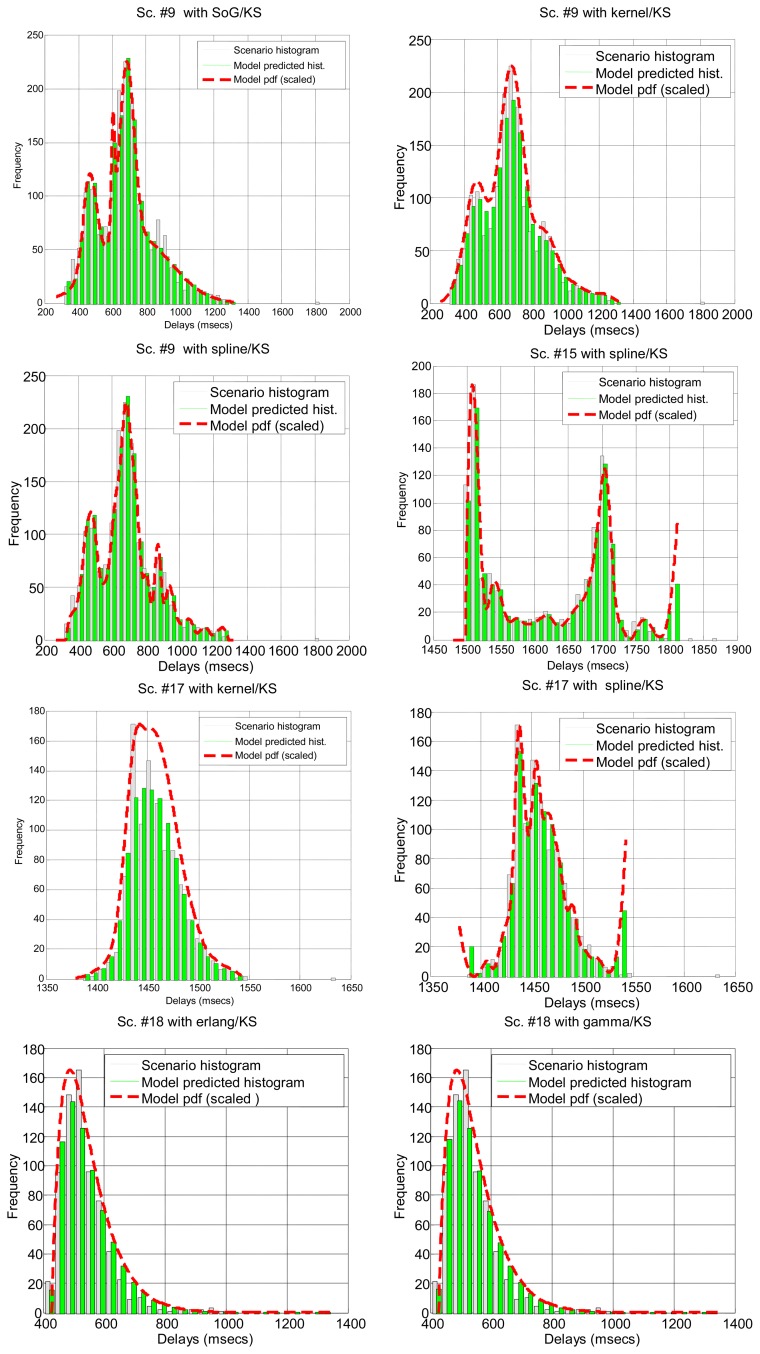

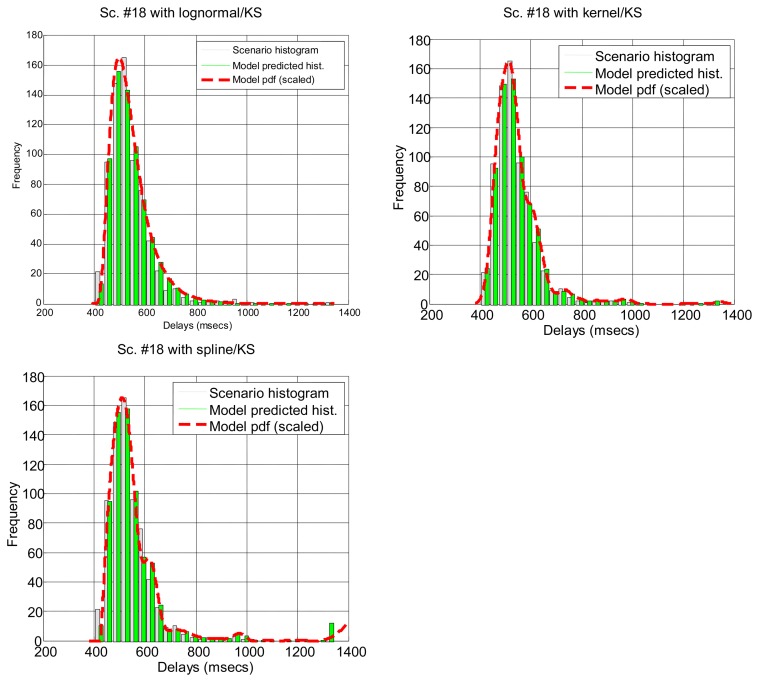
Illustrative histograms and fittings of the models that do not reject one or both hypothesis tests according to Table 4.

After running the significance tests, we see in Table 4 that, as expected from the multimodal nature of most scenarios, only a few of them can be explained by parametrical models: #3, #2 and #18 (corresponding to the B class and to a percentage of all delay values in all the scenarios below 12%). The rest of the scenarios do not support the null hypothesis. In the case of non-parametrical models, kernel and spline provide the best results, with above 40% of delay values explained. Non-rejected scenarios are displayed in Figure 5 for illustrative purposes. These results show that, for a parametrical choice, the lognormal or erlang distributions could be preferred when we are not able to detect outliers, bursts and regimes, but these are not good enough and might be applicable to very few scenarios. For a non-parametrical option, the kernel distribution should be the best election. In general non-parametrical models are able to explain scenarios from all classes (A, B, C), given their closest fitting to the particular data in the sample (which leads, on the other hand, to the previously mentioned problem of overfitting). The SoG model obtains worse results than the kernel due to its restriction in the number of modes, while the spline is also worse than the kernel due to its greater overfitting, that produces very good models of the data used for fitting but not in its goodness-of-fit with the delays reserved for hypothesis testing.

In summary, the higher percentage of non-rejected samples of all scenarios (bold numbers in Table 4) corresponds to a non-parametrical distribution, the kernel model, although with less than 50% of the total delays that have been tested. This can be further improved without filtering non-linearities in the sequence of delays, as it will be explained in the next section.

### 3.3. Results for Unprocessed Split Delays

To obtain better adjustments of the marginal models without detecting and processing bursts and regimes yet, we can divide the complete timing behaviour of the sensory loop into three different terms that are added to obtain the total delay: Server time, client time and network time. They can be modeled separately and perhaps more accurately.

These split times can be measured without much intervention in many networked systems, just by time-stamping at the client interface and at the server side the sensory data being transmitted, which neither involves a significant overload nor requires the use of distributed clock synchronization algorithms. However, this splitting is not always possible. There are a number of reasons for this: (i) the server side might not allow us to time-stamp messages because it is a closed implementation; (ii) if the format of the messages carrying the requested sensor data cannot be modified, they cannot include the time-stamps; and (iii) the clock precision on the server side might not be good enough to distinguish short server delays, which are really common—we cannot allow for zero delays in our approach because of the impossibility of many distributions to explain their occurrence: lognormal, weibull, erlang and gamma are limited to model strictly positive delays in most or all of their parameter configurations.

Due to the previous situations, we have been able to collect split information only from scenarios #4–#8, #13 and #18, summarising the results in Table 5. In general, parametric distributions do not fit better when we split the delays, except for the lognormal distribution. However, when non-parametrical distributions are set up as null hypotheses, the K-S test does not reject the null hypothesis of kernel for 66.7% of the total of delays, which is a really good result. Thus, in situations when we are not able to detect non-linearities, the kernel model should be the preferred approximation, if augmented with this simple splitting of delays.

**Figure 6.**
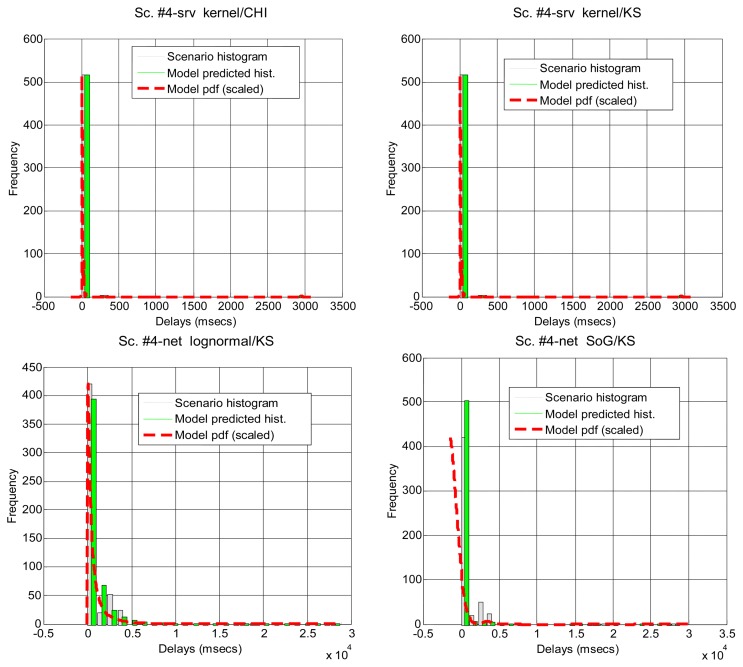

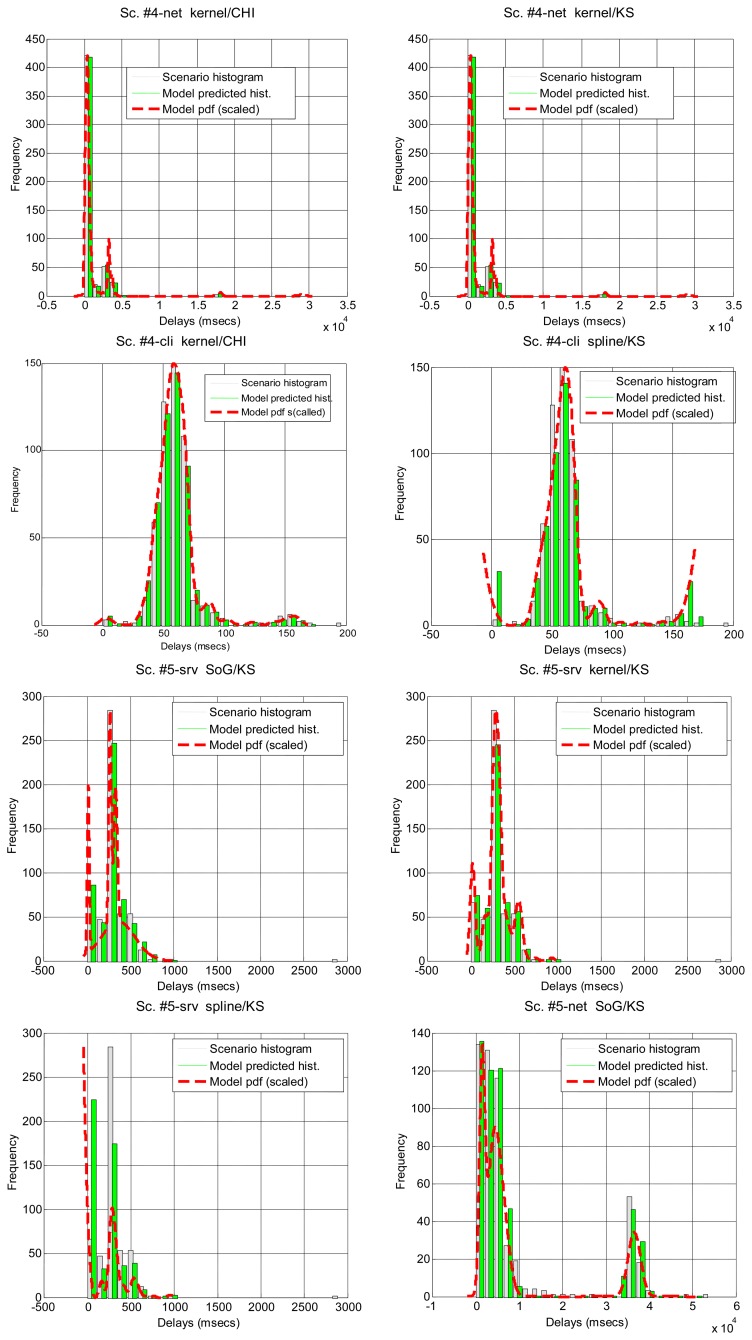

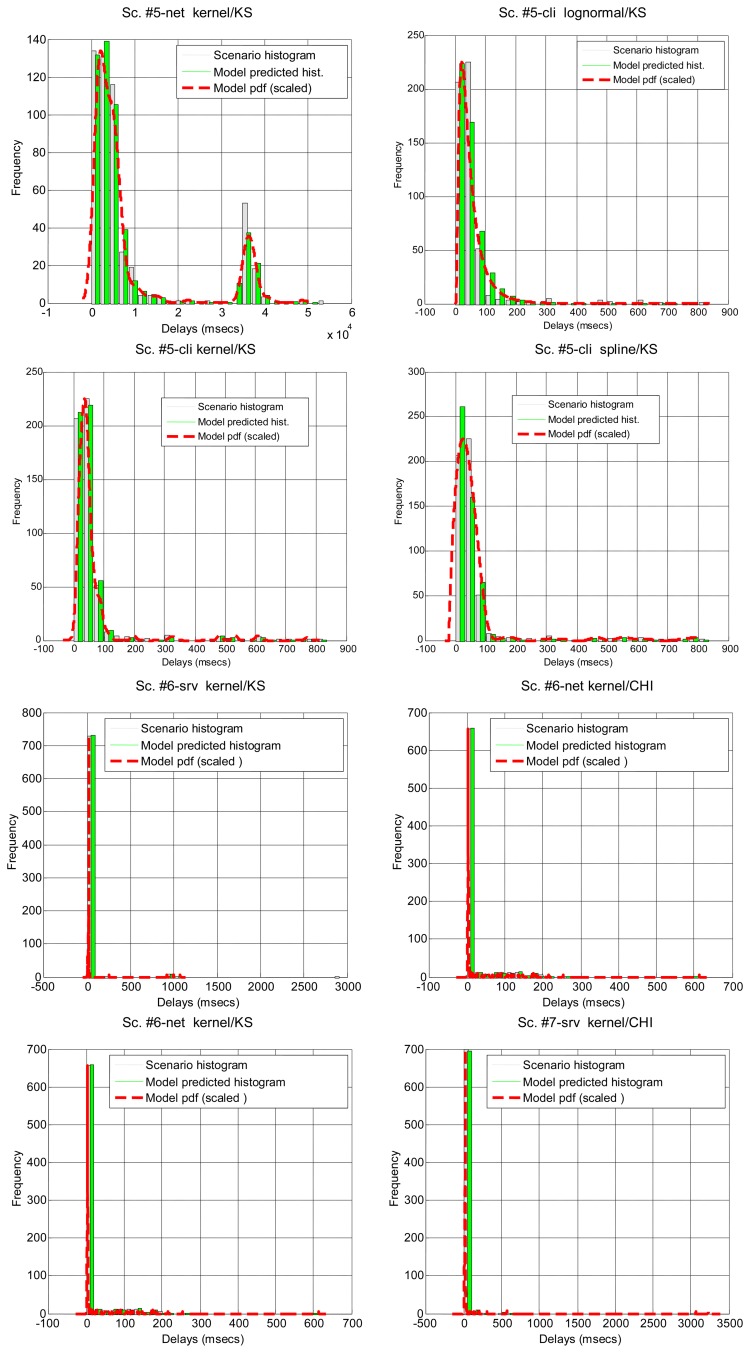

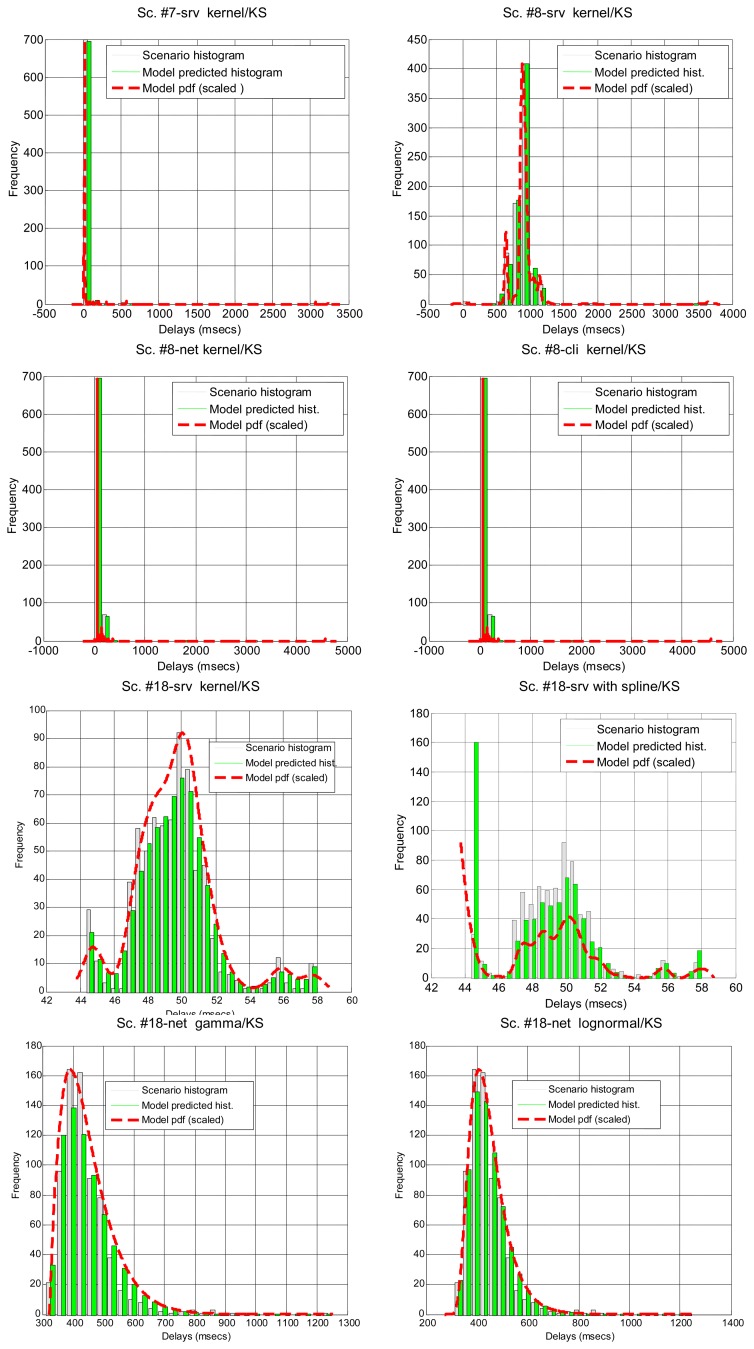

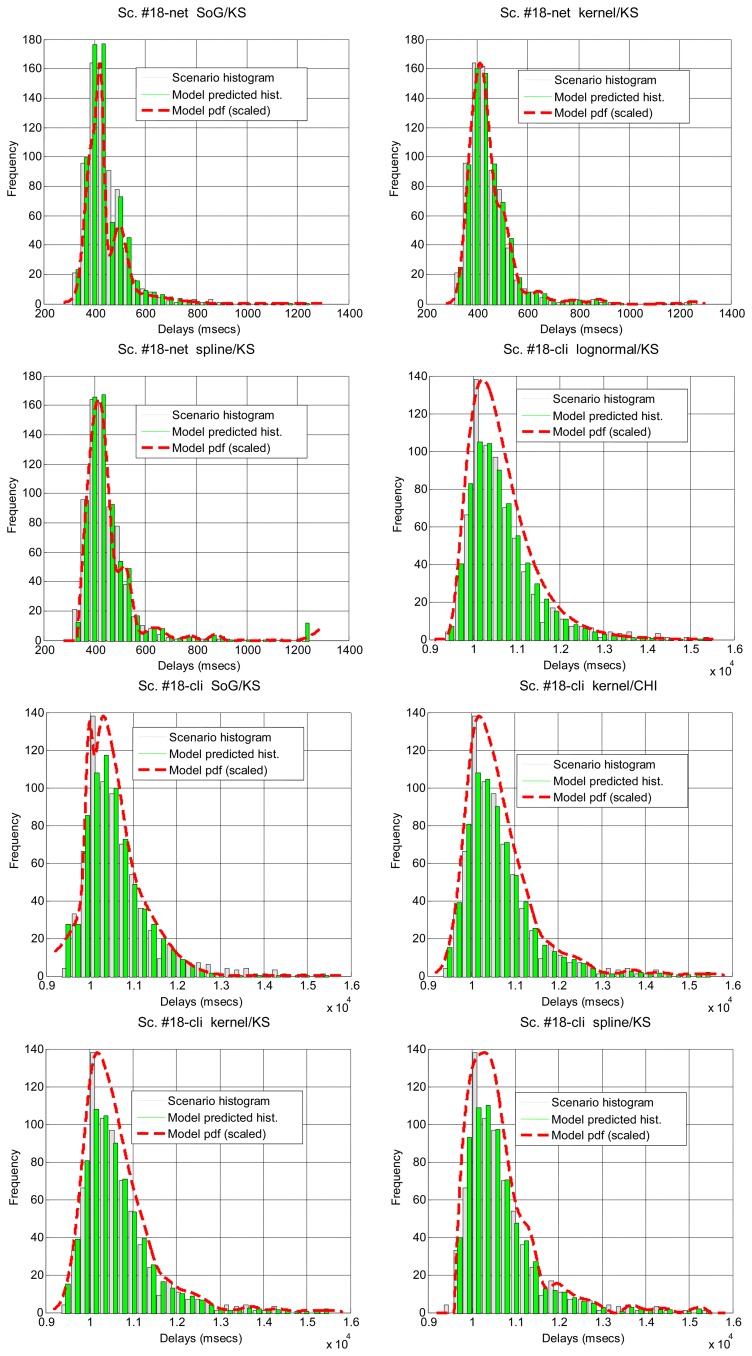
Illustrative histograms and fittings for the scenarios that can be modeled successfully after the splitting of delays (see Table 5).

In Table 5 and Figure 6 it can be seen how the different parts of the delays can be more or less predominant in the total time, and most of them can be modeled with our marginal approach. This serves to demonstrate that in a networked telerobot, taking into account only the delay produced by the network transmission of data (as many approaches in literature do) is not appropriate.

## 4. Processing Delays for the Detection of Regimes and Bursts

We have shown in Section 3 the great influence of the non-linearities of the delay signal, especially abrupt changes, in the possibility of modeling these delays with both unimodal and multimodal marginal pdfs, even when they are split into their most relevant additive components. We explore now the effects of detecting and ruling out such abrupt characteristics. In Section 4.1 we explain a method specifically designed for this work due to its accurateness/complexity trade-off, able to detect abrupt changes in the signal and therefore separate the temporal sequence into parts that do not contain those non-linearities. This method cannot guarantee, however, that the resulting segments pass the hypothesis test, only that they should be separately modeled. Section 4.2 presents its results when applied to the delays of our scenarios. Section 4.3 complements the change detection analysis through the use of a previously presented algorithm for segmentation of the signal [23] that, on the one hand, is much more involved computationally (thus, without the inclusion of especial techniques for improving computational efficiency it does not satisfies our requirement for low computational cost), but, on the other hand, tries to find only segments of the signal that can be explained by the model at hand. The results of this method are gathered in a table that serves as a near-optimal segmentation of the signal for the goal of modeling it with each probability distribution. Finally, Section 4.4 completes our analyses through the use of another two statistic tools, Q-Q graphs and probability difference graphs, which serve to draw additional conclusions on our modeling problem.

### 4.1. Efficient Abrupt Change Detection

Our approach to detect abrupt changes in the signal efficiently is based on identifying outliers. Although the precise definition of outlier is quite subjective, in our context it will be any value that is not likely to be produced by the model we have constructed up to that time: in that way we will use sequences of consecutive outliers to define abrupt changes in the underlying distribution, and thus, to separate near-stationary sequences of delays.

The literature in outlier detection is extensive (see for example [44,45]). In principle we are interested in detecting them when using both parametrical and non-parametrical models. Some outlier detection approaches could be used for that because they do not impose any particular probabilistic model for the data, for instance those that are based on clustering techniques [46,47], *i.e.*, on considering outliers those values that form small clusters separated from the main data; other approaches search for values that may be consistent with the global set of data but are not with their local neighborhoods, called spatial outliers [48]. Unfortunately, these distribution-independent techniques are usually computationally involved. Some of them are based on local distance measures, and usually designed to manage high-dimensional spaces efficiently in data-mining [49–51]. This presents another problem: they require large sets of data—long sequences of delays—which is not typically possible in our framework and the kind of scenarios found in this work. In summary, due to the lack of existing methods that are both efficient and able to deal with small samples in a non-parametrical setting, we have focused our analysis firstly on providing a solution for parametrical models only, and secondly, based on the obtained results, on deciding whether further research on non-parametrical approaches is worth. As explained later on, we report here good results with a basic method that works only with parametrical models, thus we have left for future work the study of non-parametrical ones.

The method we propose for detecting abrupt changes with low computational cost is based on the basic probability theory idea of calculating the probability that a value has not been drawn from a known model, or, in our case, that it lies in an “outlier-region” defined to be in the right-tail of the current model (by a given significance level α). The adaptation of this basic confidence interval approach to our parametrical models has provided an efficient change detection procedure that has good results even with short sequences of delays. It is described in the following.

We gather first a sequence of 40 delay values (called *blind period*) to make an initial, minimally suitable model of the data, using 20 delays for the fitting and 20 for the test. From that point on, each new delay value is considered as an outlier or not depending on the following. A fitting of the collected delays with the chosen parametrical distribution is found out, as before, with the MLE. This estimation also provides 95% confidence intervals for the parameters of the model (this is the reason why our method only works with parametrical distributions). The range of delay values that determines if the new delay is classified as an outlier or not is delimited then by the estimated offset of the distribution on the left and a bound x_α_ on the right. This x_α_, in turn, is calculated by finding the x-axis value of the chosen distribution that satisfies the predefined significance level:

(1) $$1-\text{cdf}\;(x=x_{a})=\alpha$$

We solve the above equation for *x_α_*, thus *x_α_* becomes a function of α and of the estimated parameters of the distribution, which have several possible values if we use the 95% confidence interval obtained in their estimation. In order to get a value of *x_α_* that produces as much outlier detection as possible (*i.e.*, minimizes *x_α_*), we have to select values for that available set of distribution parameters. The ones that accomplish this can be picked up by studying analytically the minimum of the equation for *x_α_*, as it is summarised in Table 6: there we have shown bi-parametrical distributions, because the fitting process estimates firstly the offset (without 95% confidence interval) and, through a bi-parametrical fitting tool of Matlab, the rest of the parameters and their 95% confidence intervals. Exceptionally, for the case of the lognormal distribution, we also have the asymptotic variance of the offset [52], and thus, a 95% confidence interval for that parameter too.

If the new delay value lies outside the calculated range, it should be treated as an outlier. If this situation persists for five to forty consecutive outliers, all of them are discarded from the scenario as a burst. Forty consecutive outliers indicate a change of regime. If that occurs, these forty delays will be included in the beginning of the new regime, *i.e.*, in a new blind period. Finally, isolated—singleton—outliers will be treated as part of the current distribution, but if the consecutive number of outliers is higher than one and less than five, these outliers will be discarded.

### 4.2. Results for Processed Delays

In Table 7 we show how the confidence interval method explained before produces better fits than the splitting of delays (recall Table 5) in all parametrical models. This method, with these parametrical distributions, explains well classes A and B (it does not reject the null hypothesis of the tests), especially when we use the lognormal, including the mostly deterministic scenario #13 corresponding to the Arduino system. As it already happened with split data, erlang suffers both from split and change detection, which, along with their intrinsic reduction in the size of the sample, are procedures that call for more flexible models (remember that one of the parameters of the erlang is forced to be a natural number; the gamma, which is a generalization of the erlang, does not have that limitation). Notice that our previous categorisation {A, B, C} is reflected in these final results: scenarios of class B (they have no trend) are all explained by the methods, even with models that have low rate of success with the data of other classes, while classes A and C are more problematic (but the lognormal is able to model many scenarios from class A, whose trends are successfully reduced by the confidence interval method). It is observed again that the χ^2^ test provides less model adjustments than the K-S due to its sensitivity to the reduction of sample size produced by both splitting and change detection methods.

**Figure 7.**
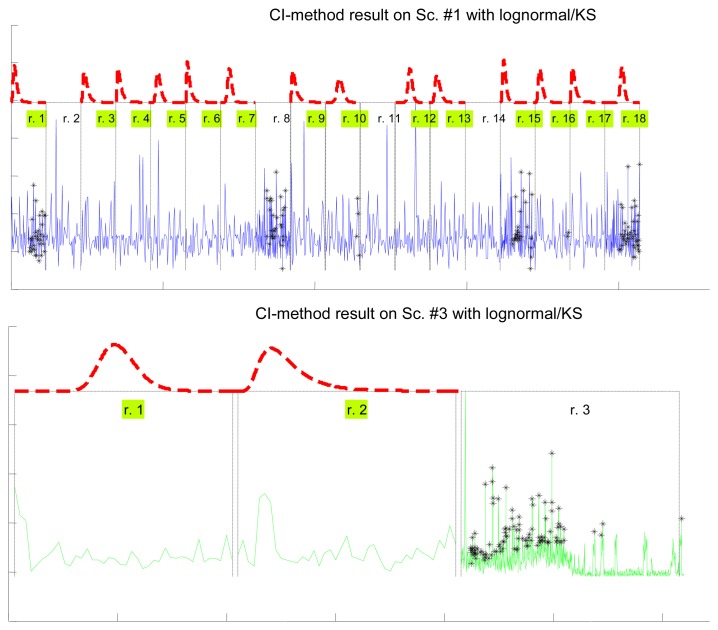

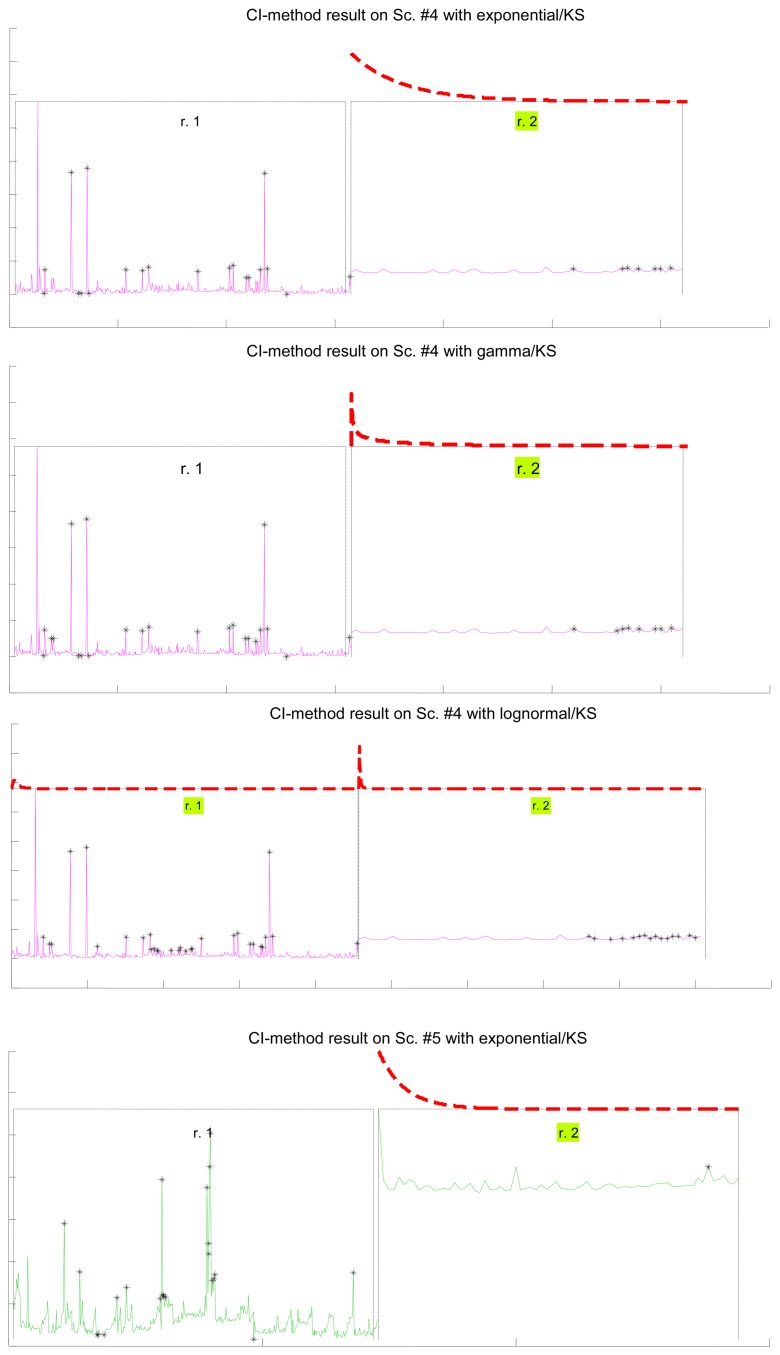

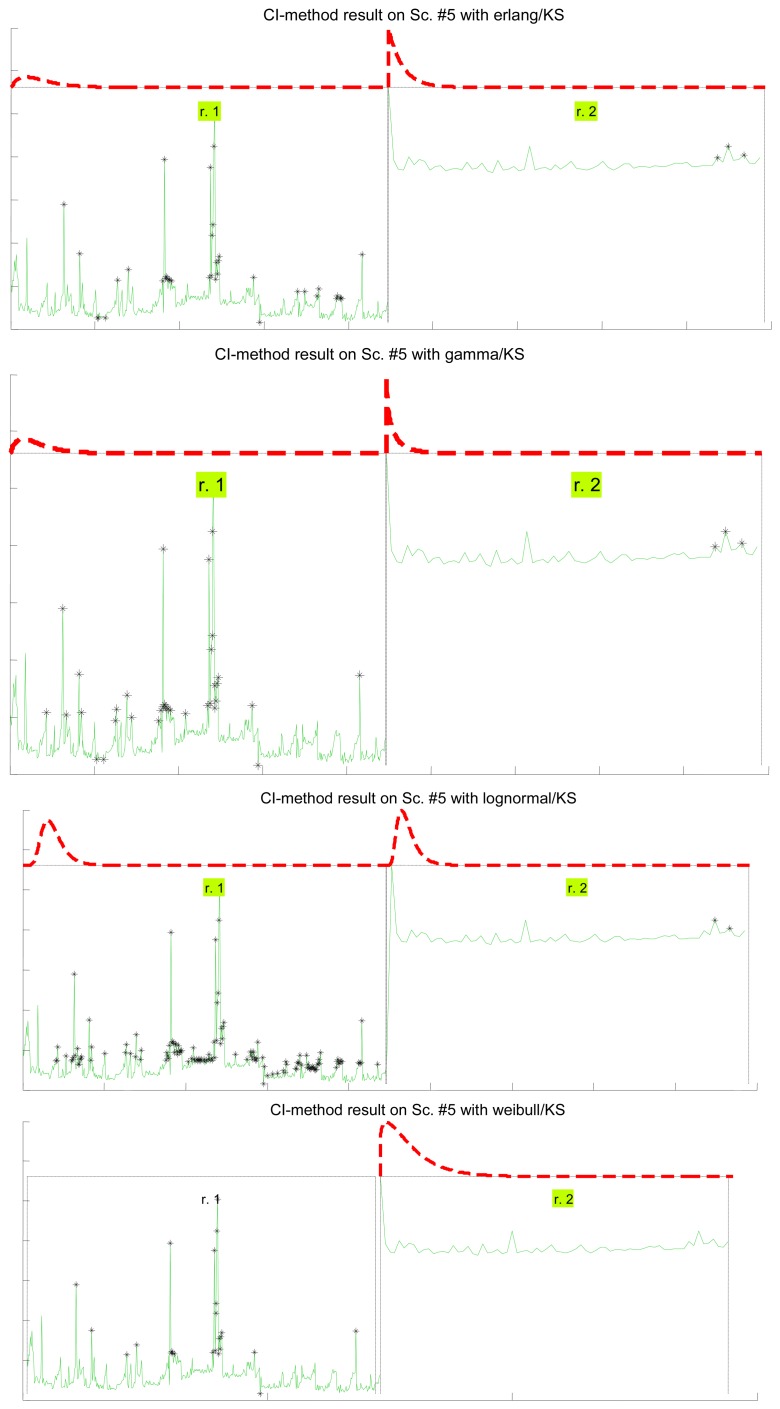

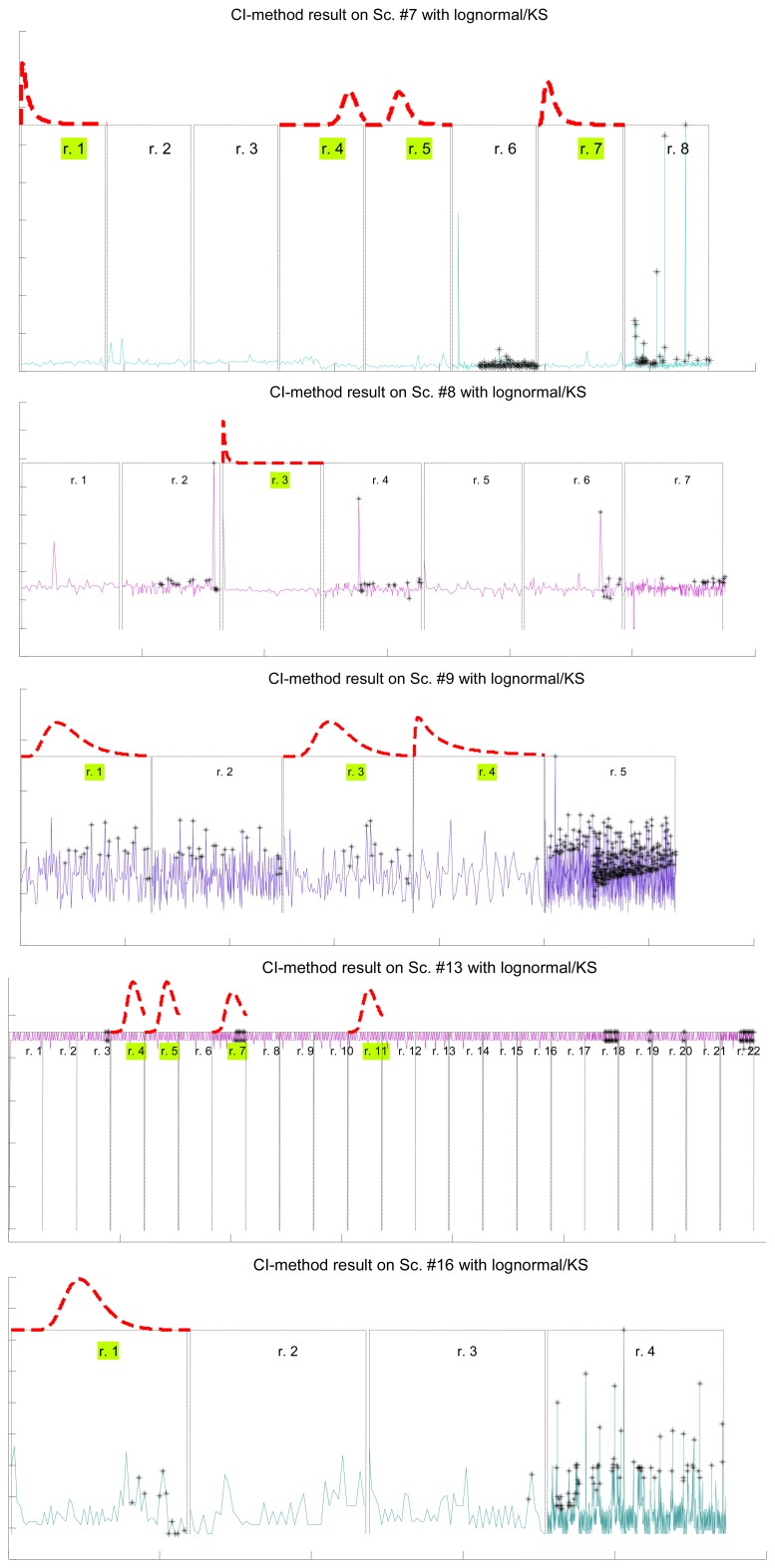

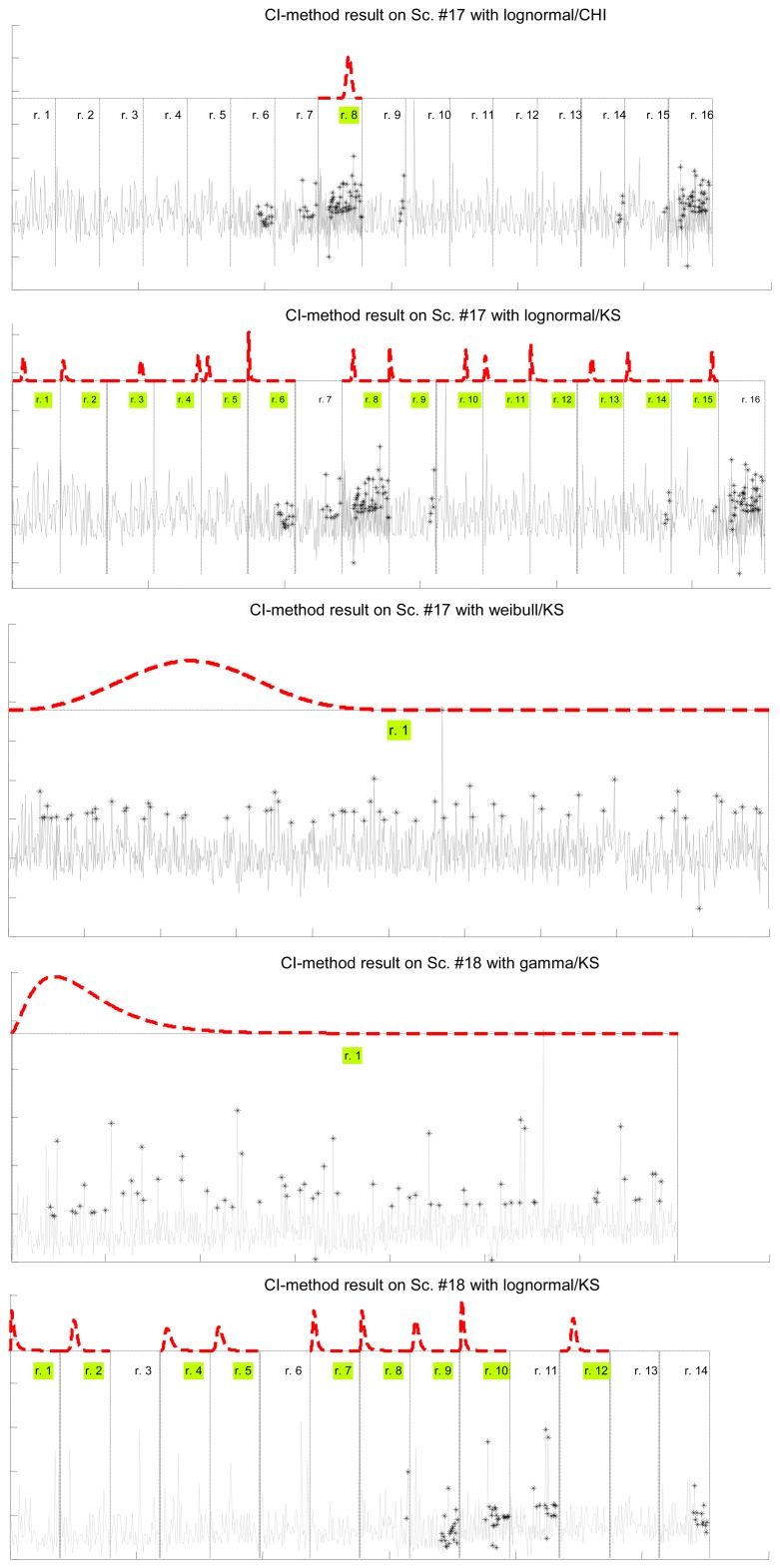
Plots of the detection of outliers, bursts and regime changes by our method of confidence intervals (CI) with different parametrical distributions. Black asterisks correspond to the detected bursts and outliers. All the models successfully found for the regimes detected by the algorithm (red lines over the green-marked segments) are depicted with the same aspect ratio in order to provide a fair comparison of their shapes. The delays below them have been stretched accordingly.

Figure 7 illustrates the results for the scenarios that have been modeled successfully. On the top plot of each figure we have drawn the models found for the different segments (regimes) that have been separated by the confidence interval method. Bursts and outliers are marked with black asterisks.

### 4.3. Near-Optimal Abrupt Change Detection

In this section we include the results obtained when we use a different change detection algorithm, reported elsewhere as the *stateless* algorithm [23], to separate the delay sequence into almost stationary segments. The algorithm, that we will call here the near-optimal method, relies in executing the complete hypothesis testing each time a new delay value is gathered from the system, which makes its computational burden much higher than the one of the method introduced in Section 4.1. Therefore, this one is included here more as a limit of the results that any detection algorithm can provide with our marginal distribution approach than as an alternative to be implemented in very low computational power system.

The algorithm is quite intuitive. Essentially, it firstly makes up a regime of a minimum size (we use 40 in this work to be in the same conditions as the confidence interval method); when such a minimal regime is collected, it uses the hypothesis test: a rejection moves that regime one delay value on—*i.e.*, we request new sensory data and obtain a new delay measurement—forgetting the oldest delay value of the current regime, while a non-rejection makes the algorithm to try to enlarge the regime with the next delay values, until one is added that makes the test to reject it; when such an enlarged regime is finally rejected, its longest non-rejected portion is kept as a definitive regime of the scenario. It is clear that with this algorithm it is very likely to obtain segments of the signal that can be explained statistically by the model at hand, since they are constructed specifically for that purpose. The algorithm does not find such segments when the scenario is not compatible with the model.

The results are shown in Table 8. Notice how the pattern already found with the confidence interval method, and even with unprocessed scenarios or split delays, appears here again: the lognormal model is the most successful one (although in this case all the others have a very good behaviour as well), both in the number of scenarios explained successfully and in the goodness of the models obtained, *i.e.*, the *p*-values.

### 4.4. Additional Analysis of Processed Delays

Apart from assessing the fitting of parametrical and non-parametrical models with hypothesis tests, we have also analysed in this work, with other statistical tools, the modeling results when using the confidence interval method: Quantile-Quantile plots and Probability Difference Graphs. They complete our statistical study of the problem, providing additional conclusions that are explained in the following:

#### Quantile-Quantile (Q-Q) plot

The quantile-quantile (Q-Q) plot is a graph of the input (observed) data values against the theoretical (fitted) distribution quantiles. Both axes of this graph are in units of the input data set. Q-Q plots can be used to determine, qualitatively, the goodness-of-fit of the models, but also other interesting questions such as how heavy their right tails are, which has consequences when used for predicting future time delays: a model that has heavier tail (greater area in the right tail) than the data that it is modeling will report a smaller probability of closing the sensory loop before the given time requirement than actually exists. That will produce pesimistic predictions, *i.e.*, longer expected delays, which can be useful in teleoperation [53]: in the case that there is no model good enough for representing the data tightly at all quantiles, it should be better to be pessimistic, *i.e.*, to work with the model that has heavier tail.

**Figure 8.**
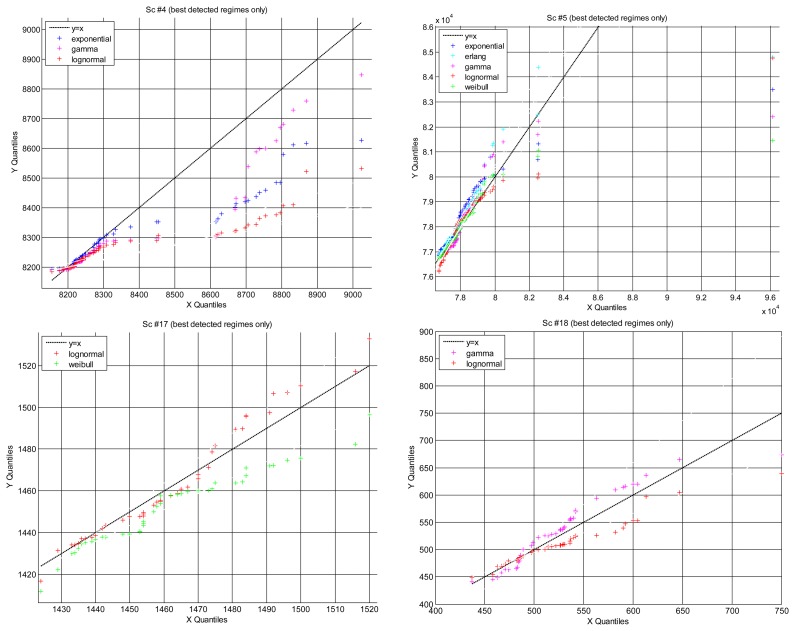
Q-Q plots for those regimes obtained by the confidence interval method where more than one parametrical model explains successfully the data obtaining their best *p*-values. The abscissas correspond to the data quantiles, while the ordinates are the models.

Figure 8 represents the Q-Q plot of all scenarios that we have found where different models have explained well the same segments of data (the only case when we can carry out a fair comparison). In general, all scenarios are better modeled in the lower quantiles, *i.e.*, the right tails of the distributions are not good models of the data, which is natural because there is less delays gathered in those tails. We can also observe that data are better modeled in scenarios #17 and #18 than in scenarios #4 and #5, but note that this is only a qualitative appreciation: all the depicted cases have passed the hypothesis test, therefore they are all statistically good as models of the data.

In that figure we see that Q-Q plots for scenarios #17 and #18 fit quite well the data. In contrast, scenarios #4 and #5 are arced, or “S” shaped, indicating worse models (although still within the acceptance of the hypothesis test). Also, all models there become more dispersed than the data in the highest quantiles, *i.e.*, in the right tails. In particular, the lognormal is the most heavy tailed model, and exponential/gamma the less heavy tailed. In these scenarios, where the models do not fit the data as well as in others, the lognormal distribution would predict less probability to close the sensory loop in a shorter time than the actual one, therefore being more conservative for doing control than the others. As explained before, being conservative is an interesting quality in our problem, since it facilitates to satisfy strict safety requirements with higher probability.

#### Probability Difference Graph (PDG)

**Figure 9.**
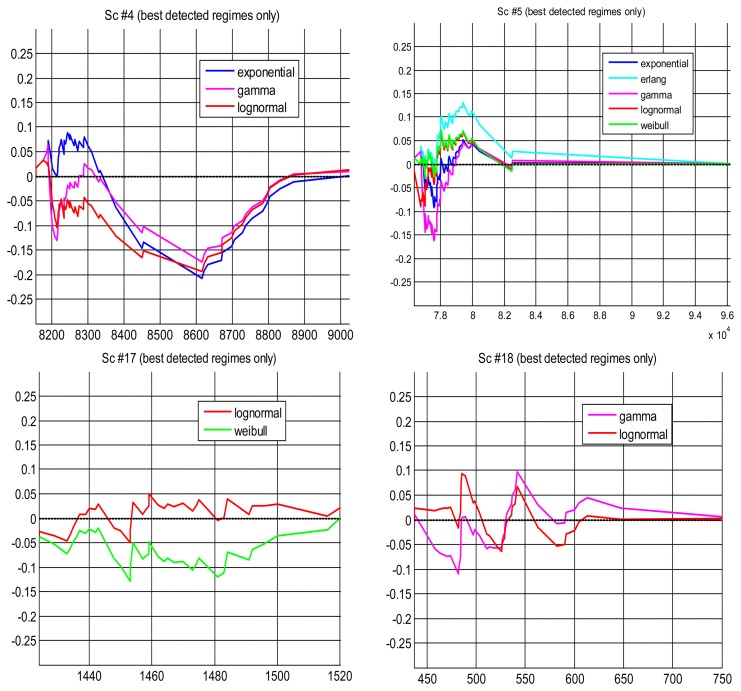
PDGs for those regimes obtained by the confidence interval method where more than one parametrical model explain successfully the data obtaining their best *p*-values.

The probability difference graph (Figure 9) is a plot of the difference between an empirical cumulative distribution function (cdf) and a theoretical cdf. This graph can be used to put in order the theoretical distributions in case we have to choose a model, since it yields a qualitative measure of distance between them. It also provides a general, qualitative indication of their goodness to fit the data: the closest to the zero horizontal, the better the fitting. Although all PDGs are for models that have been successful in modeling scenarios (according to the hypothesis test, reflected here in the small values of the curves in the ordinate axis), we can observe in the figure that in some cases the modeling has been better than in others. As happened in the Q-Q plots, scenario #4 has produced the worse fittings, which is reflected in the pronounced belly of all model curves around the middle of the delay values: if they are used for prediction they will be conservative in that area, which could be an interesting property as explained before. In that particular scenario, the gamma distribution seems slightly better than the other two, but their difference is rather small. In scenario #5 we obtain a pdf farther from zero when the erlang model is chosen as null hypothesis. In scenario #17 the lognormal has been better than the weibull. In scenario #18 the lognormal is also better for higher values of the delays, which is important in prediction.

## 5. Conclusions and Future Work

This paper has shown how the time delay of the sensory data flow for a networked telerobot system, *i.e.*, the time that passes since a sensor request is issued in the client until the data is received and processed, has an interesting feature that allows us to model it in a minimalistic way: when abrupt changes in the delay sequence are properly detected, the remaining segments are mostly stationary and *iid.* Based on that result, we have presented a thorough statistical characterization of these sequences of delays, based on both parametrical and non-parametrical probability density functions that model marginal distributions of the delays. When applied to sequences of delays that have not been processed for detecting those abrupt changes, they do not show as particularly useful (with the exception of some non-parametricals), but we can improve those initial results firstly by splitting the delays into their natural additive components and then by applying a new method to detect non-linearities such as abrupt changes of regimes, outliers or bursts. The results reported in this paper can be summarised, qualitatively, as follows:

- Unprocessed scenarios can only be reasonably well modelled with non-parametrical distributions. However, their use must be carefully considered, since they are more computational involved and prone to overfitting, *i.e.*, they model too well the already gathered delays at the expense of not modeling so well the delays to come just afterwards.
- Even when scenarios are unprocessed, the lognormal model performs better or equal than other parametrical ones. The kernel model is clearly better than the others in the case of non-parametrical models.
- If splitting the delays into three natural components (server + network + client delays) is possible, the performance of the lognormal model increases appreciably, while other parametrical models do not do so well. The kernel model also increases remarkably its performance with respect to other non-parametrical models, reaching near 70% of scenarios being explained. Therefore, kernel model could be the choice in that situation (again considering carefully the overfitting problem).
- Our simple confidence interval method for detecting abrupt changes in the delay sequence (only applicable to parametrical models) increases the performance of the lognormal as in the case of splitting the delays, but, in addition, it widens the applicability of the model, being able to explain scenarios of classes A, B and C (unlike in the splitting delays case). When there are relevant limitations in the computational cost available to execute on-line the change detection procedure, this method along with the lognormal should be the choice in general applications of the marginal modeling approach.
- As shown in the Q-Q plots, when using the confidence interval method, exponential and gamma models do not provide as good results as the lognormal, and in complicated scenarios that cannot be modeled well anyway, the latter could be a reasonable choice since it provides more conservative (pessimistic) estimates of the delays, which can serve to guarantee better the time requirements of the system.
- The chi-squared hypothesis test is not as good to detect whether a model explains well the data as the Kolmogorov-Smirnov test, mainly due to its sensitivity to short samples.

In this paper we have also identified intuitive categories of scenarios that cover most existing combinations of features that can appear in these sequences of delays (classes A, B and C). Just to link very briefly this classification to the actual results of our analyses, according to Section 3.3, when the scenarios are not processed for change detection, some scenarios from class B can be explained by parametrical models, which agrees with the fact that no abrupt changes with long lasting different regimes are present (Table 4); however the same models are not able to explain the rest of scenarios, which leads to a not very good utility of the unprocessing approach (this does not change even when delays are split into three additive components—network, server, client—, as shown in Table 5, which demonstrates that the features of the total delay signal are present in all of them, although the number of scenarios of class B that can be explained now is greater). Table 7 shows the modeling results when abrupt change detection is included through our confidence interval algorithm: then, classes B1 and B2 can be explained, as well as class A because the algorithm detects the accumulation of smooth trend fluctuation during certain time as an abrupt change at the end of that time, making the total fluctuation in each of these parts of the signal much smaller than in the original unprocessed scenario. Class C is more difficult to model, but the lognormal is able to explain some of those scenarios as well, including a largely deterministic one (scenario #13), included in our study to test the limits of stochasticity in our approach.

In the future we plan to analyse further this characterization problem (in particular, to devise new computationally efficient change detection algorithms that approach better the results of the near-optimal procedure explained in Section 4.3), and also to use these results to implement on-line dynamic estimators of the time delay, which will be the basis for a control of the sensor density that allows us to control in turn the networked telerobot in real-time as optimally as possible. In such environment, we will also develop methods for using more than one modeling procedure concurrently, aimed at guaranteeing the system performance in the minority of cases where the *iid* assumption cannot be satisfied.

## Figures and Tables

**Table 1.** Summary of the experimental scenarios that have been set up for modeling the delay in sensory data transmission from our networked telerobots (more details on the elements of this table can be consulted in the main text).

**Scenario**	**Robot**	**Sensor**	**Resolution**	**Location**	**Client Computer**	**Server Computer**	**Network**
#1	SANCHO	Laser	181 data points	Same building	A	a	1
#2	SANCHO	Webcam	20% full B&W	Same city	B	a	2
#3	SANCHO	Webcam	40% full B&W	Same city	B	a	2
#4	SANCHO	Webcam	30% full B&W	Same city	B	b	3
#5	SANCHO	Webcam	100% full color	Same city	B	b	3
#6	SANCHO	Webcam	10% full B&W	Same building	A	b	4
#7	SANCHO	Webcam	50% full B&W	Same building	A	b	4
#8	SANCHO	Webcam	100% full B&W	Same building	A	b	4
#9	SURVEYOR	Webcam	5.86% full color	Same building	C	c	5
#10	GIRAFF	Odometry	9323 bytes	Same building	D	d	1 + 4
#11	PHP simulated odometry only	Null (echo)	20 bytes	Ituzaingó (Argentina) & Málaga (Spain)	E	e	4 + 6 + 7
#12	C++ mobile robot simulator	Odometry	80 bytes	Same city	F	e *	2+4
#13	Arduino	Analog input	27 bytes	Same desk (p2p)	G	f	8
#14	PHP simulated complete robot	Null (echo)	4 bytes	Same building	H	h	4
#15	PHP simulated complete robot	Simulated camera	100% RGB	Same computer	H	g	No net
#16	PHP simulated complete robot	Simulated laser	2048 data points	Same building	H	h	4
#17	PHP simulated complete robot	Simulated camera	100% RGB	Same computer	H	h	4
#18	PHP simulated complete robot	Simulated camera	100% RGB	Plymouth (UK) & Málaga (Spain)	I	e	4 + 6 + 7

*Legend*: A: Pentium M@1.8 GHz, 1 GB RAM, LINUX, FireFox. B: Intel Core Duo T7200@2 GHz, 2 GB RAM, LINUX, FireFox. C: Android smartphone Galaxy SII. D: Desktop PC Intel R CoreTM i5 3330@3 GHz, 8 GB RAM, Windows XP, teleoperation graphical interface of [24]. E: Laptop Intel core 2 duo T6400@2 GHz, 2 GB RAM, Windows XP. F: Laptop Intel core 2 duo T7200@2 GHz, 2 GB RAM, Linux. G: Desktop PC Intel core i7 960@3.20 GHz, 12 MB RAM, Linux. H: Desktop PC Intel core i7 940@2.93 GHz, 12 GB RAM, Linux. I: Laptop Intel Core 2 Duo P9600@2.53 GHz, 4 GB RAM, Linux. a: Intel Pentium M@2 GHz, 1 GB RAM, LINUX. b: Intel Pentium IV@3.2 GHz, 1 GB RAM, WINDOWS. c: Surveyor, Android. d: Mini-PC, Intel core i3 M@2.10 GHz, 3 GB RAM, Windows Embedded Standard. e: Desktop PC Intel core i7 950@3.07 GHz, 16 GB RAM, Linux. f: Arduino board with Atmel Atmega 328P@16 MHz, 2 KB RAM, no OS. g: Desktop PC Intel core i7 940@2.93 GHz, 12 GB RAM, Linux. h: Mini PC Intel core i5 650@3.20 GHz, 4 GB RAM, Windows 7 Enterprise N. 1: WiFi 802.11b/g, local University provider in the same building as the robot. 2: WiFi 802.11b/g, ISP provider located in Madrid (about 600 km from our lab). 3: RTC 56 Kbps, ISP provider located in Madrid (about 600 km from our lab). 4: Twisted-pair 1GBs Cable. 5: WiFi *ad-hoc*. 6: Submarine data cables [25]. 7: WiFi 802.11b/g ISP provider in Ituzaingó, Argentina. 8: USB 2.0 point-to-point cable.

* robot simulation running in a virtual machine with Windows XP and the BABEL development system [26].

**Table 2.** Two networked telerobots (one non-mobile and the other mobile) as examples of the usual kind of components that are found in these systems.

**Project**	**Physical Structure**	**Sensors**	**Communications**	**Software**	**Interface**
The Mercury Project	IBM SR5427 robotic arm by Sankyo	CCD Camera gray 192 × 165	Internet, Ethernet card, serial line	Server A: Sun SPARCServer 1000, SunOS 5.3 Server C: PC, MS-DOS	Camera image X,Y,Z coordinates Robot Movement (low-level commands)

Xavier	B24 mobile base by Real World Interface	Bump panels Wheel Encoders 24 sonar ring 30° fov laser Color camera + pan-tilt Speaker Speech-to-text card	Wireless Ethernet Thin-wire Ethernet	Two PC computers and a 486 laptop, all of them running Linux Sparc5 workstation with Netscape webserver	Camera image Zoomable map Robot Movement (high-level commands)

**Table 3.** Mathematical forms of the parametrical pdfs used in our characterization. All of them are considered three-parametrical, being γ the offset, *i.e.*, the lower bound for the delays.

**pdf**	**Constraints**
$\text{Erlang}(x;\alpha ,\beta ,\lambda )=\frac{{\left(x-\gamma \right)}^{\left(\alpha -1\right)}}{\beta^{\alpha }(\alpha -1)!}e^{\frac{-(x-\gamma )}{\beta }}$	*x*, *α*, *β*, *γ* ∈ ℝ *x*, *α*, *β*, *γ* > *0*
$\text{Gamma}(x;\alpha ,\beta ,\lambda )=\frac{{\left(x-\gamma \right)}^{\left(\alpha -1\right)}}{\beta^{\alpha }\Gamma (\alpha )}e^{\frac{-(x-\gamma )}{\beta }}$	*x*, *α*, *β*, *γ* ∈ ℝ *x*, *α*, *β*, *γ* > *0*
$\text{Lognormal}(x;\mu ,\sigma ,\gamma )=\frac{1}{(x-\gamma )\sigma \sqrt{2\pi }}e\frac{-{\left(\ln(x-\gamma )-\mu \right)}^{2}}{2\sigma^{2}}$	*μ*, *γ* ∈ ℝ *x*, *σ*^2^ ≥ 0
$\text{Weibull}(x;k,\lambda ,\gamma )=\frac{k}{\lambda }{\left(\frac{x-\gamma }{\lambda }\right)}^{k-1}e^{-{\left((x-\gamma )/\lambda \right)}^{k}}$	*λ*, *k*, *γ*, *x* ≥ 0
*Exponential*(*x*; *λ*, *γ*)=*λe*^−^*^λ^*^(^*^x^*^−^*^γ^*^)^	*λ*, *γ*, *x* ≥ 0

**Table 4.** Non-rejected hypotheses for each unprocessed scenario, indicating which class it belongs to and the percentage of non-rejected delay values (in bold), that is, the proportion of delays explained by that model with respect to all delay values of all scenarios tested. This percentage is calculated independently of the length of each scenario for a fairer comparison.

	**Exponential** **(*p*-Value)**	**Erlang** **(*p*-Value)**	**Gamma** **(*p*-Value)**	**Lognormal** **(*p*-Value)**	**Weibull** **(*p*-Value)**	**SoG** **(*p*-Value)**	**Kernel** **(*p*-Value)**	**Spline** **(*p*-Value)**
χ^2^	-	-	-	-	-	-	#4B2(0.14) #7B2(0.51) #8C(0.16) **16.7%**	-

KS	-	#3A/B2(0.03) #18B1(0.06) **11.1%**	#18B1(0.08) **5.6%**	#2B1(0.07) #18B1(0.03) **11.1%**	#3A/B2(0.03) **5.6%**	#2B1(0.13) #3A/B2(0.05) #5B2(0.04) #8C(0.39) #9A(0.12) **27.8%**	#2B1(0.30) #4B2(0.44) #5B2(0.21) #7B2(0.05) #8C(0.50) #9A(0.04) #17A(0.07) #18B1(0.40) **44.4%**	#3A/B2(0.28) #9A(0.25) #15A(0.03) #17A(0.18) #18B1(0.52) **27.8%**

**Table 5.** Condensed comparative table of non-rejected scenarios for both unprocessed (U) and split (S) scenarios, with parametrical and non-parametrical distributions (the unprocessed part is the same as Table 4).

	**Exponential** **(*p*-Value)**	**Erlang** **(*p*-Value)**	**Gamma** **(*p*-Value)**	**Lognormal** **(*p*-Value)**	**Weibull** **(*p*-Value)**	**SoG** **(*p*-Value)**	**Kernel** **(*p*-Value)**	**Spline** **(*p*-Value)**
χ^2^ **(U)**	-	-	-	-	-	-	#4B2(0.14) #7B2(0.51) #8C(0.16) **16.7%**	-

KS **(U)**	-	#3A/B2(0.03) #18B1(0.06) **11.1%**	#18B1(0.08) **5.6%**	#2B1(0.07) #18B1(0.03) **11.1%**	#3A/B2(0.03) **5.6%**	#2B1(0.13) #3A/B2(0.05) #5B2(0.04) #8C(0.39) #9A(0.12) **27.8%**	#2B1(0.30) #4B2(0.44) #5B2(0.21) #7B2(0.05) #8C(0.50) #9A(0.04) #17A(0.07) #18B1(0.40) **44.4%**	#3A/B2(0.28) #9A(0.25) #15A(0.03) #17A(0.18) #18B1(0.52) **27.8%**

χ^2^ **(S)**	-	-	-	-	-	-	#4srvB2(0.87) #4netB2(0.47) #4cliB2(0.06) #6netB2(0.33) #7srvB2(0.08) #18cliB1(0.07) **28.6%**	-

KS **(S)**	-	-	#18netB1(0.05) **4.8%**	#4netB2(0.04) #5cliB2(0.03) #18cliB1(0.06) #18netB1(0.07) **19%**	-	#4netB2(0.17) #5srvB2(0.17) #5netB2(0.31) #18cliB1(0.05) #18netB1(0.22) **23.8%**	#4srvB2(0.23) #4netB2(0.37) #5srvB2(0.24) #5netB2(0.12) #5cliB2(0.80) #6srvB2(0.07) #6netB2(0.60) #7srvB2(0.42) #8srvC(0.48) #8netC(0.17) #8cliC(0.17) #18cliB1(0.03) #18netB1(0.50) #18srvB1(0.24) **66.7%**	#4cliB2(0.13) #5srvB2(0.58) #5cliB2(0.05) #18cliB1(0.04) #18netB1(0.59) #18srvB1(0.73) **28.6%**

**Table 6.** Confidence interval parameter selection that minimizes x_α_ depending on each parametrical distribution assumption.

**Distribution (bi-parametrical; the offset has no 95% c.i.)**	**Parameters provided by estimation, with 95% c.i.**	**x_α_**	**Confidence interval parameter selection** * that minimizes x_α_
*Erlang*(*x*; *α*, *β*)	a_L_, a_U_, b_L_, b_U_	$b\gamma_{\text{inc}}^{-1}((1-\alpha )(a-1)!/\Gamma (a),a)$	a_L_, b_L_
*Gamma*(*x*, *α*, *β*)	a_L_, a_U_, b_L_, b_U_	$\mu \gamma_{\text{inc}}^{-1}((1-\alpha )(k-1)!/\Gamma (k),k)$	a_L_, b_L_
*Longnormal*(*x*; *μ*, *σ*)	μ_L_, μ_U_, σ_L_, σ_U_	$e^{\mu -\beta \sigma \sqrt{2}}$	$if\{\begin{array}{c} \beta \leq 0,\mu_{L},\sigma_{L}\\ \beta >0,\mu_{L},\sigma_{L} \end{array}$
*Weibull*(*x*; *k*, *λ*)	k_L_, k_U_, λ_L_, λ_U_	*λ*(−ln *α*())^1/^*^k^*	λ_L_, k_U_
*Exponential*(*x*; *λ*)	λ_L_, λ_U_	− *λ* ln(*α*)	λ_L_

* *β* = *erfcinv*(2(1 − *α*)), where erfcinv is the inverse of the complementary error function. $\gamma_{\text{inc}}^{-1}$ is the gamma incomplete inverse function.

**Table 7.** Comparative table of non-rejected scenarios for unprocessed (U), split (S) and confidence interval method (C) with parametrical distributions (unprocessed and split values are copied from tables 4 and 5 respectively).

	**Exponential** **(*p*-Value)**	**Erlang** **(*p*-Value)**	**Gamma** **(*p*-Value)**	**Lognormal** **(*p*-Value)**	**Weibull** **(*p*-Value)**
χ^2^ **(U)**	-	-	-	-	-

KS **(U)**	-	#3A/B2(0.03) #18B1(0.06) **11.1%**	#18B1(0.08) **5.6%**	#2B1(0.07) #18B1(0.03) **11.1%**	#3A/B2(0.03) **5.6%**

χ^2^ **(S)**	-	-	-	-	-

KS **(S)**	-	-	#18netB1(0.05) **4.8%**	#4netB2(0.04) #5cliB2(0.03) #18cliB1(0.06) #18netB1(0.07) **19%**	-
χ^2^ **(C)**	-	-	-	#17A(0.17) **0.7%**	-

KS **(C)**	#4B2(0.19) #5B2(0.47) **1.4%**	#5B2(2;0.09) **5.1%**	#4B2(0.45) #5B2(2;0.32) #18B1(0.15) **10.7%**	#1A(14;0.27) #3A/B2(2;0.35) #4B2(2;0.30) #5B2(2;0.44) #7B2(4;0.28) #8C(0.57) #9A(3;0.30) #13C(4;0.08) #16B2(0.10) #17A(14;0.44) #18B1(9;0.50) **23.5%**	#5B2(0.42) #17A(0.17) **6%**

**Table 8.** Results obtained with the near-optimal change detection algorithm. The items marked in bold have the best p-value of their corresponding row, *i.e.*, they are the best models found for that scenario.

	**Exponential** **(*p*-Value)**	**Erlang** **(*p*-Value)**	**Gamma** **(*p*-Value)**	**Lognormal** **(*p*-Value)**	**Weibull** **(*p*-Value)**
χ^2^	-	-	-	**-**	-

KS	**#1A(14;0.08)** #2B1(4;0.06) #3A/B2(11;0.04) #4B2(6;0.06) **#5B2(3;0.51)** #6B2(10;0.08) #7B2(10;0.03) #8C(12;0.12) #9A(22;0.04) #10B1(15;0.07) #11C(12;0.04) #12A(12;0.06) - #14C(0.03) #15A(12;0.04) #16B2(13;0.05) #17A(12;0.12) #18B1(9;0.03) **85.5%**	#1A(15;0.07) #2B1(6;0.11) #3A/B2(11;0.04) **#4B2(8;0.24)** #5B2(4;0.20) **#6B2(10;0.19)** #7B2(12;0.06) #8C(13;0.12) #9A(26;0.05) #10B1(15;0.05) **#11C(13;0.09)** **#12A(12;0.071)** #13C(0.038) #14C(4;0.03) #15A(17;0.04) **#16B2(14;0.13)** #17A(13;0.14) #18B1(12;0.09) **78%**	#1A(12;0.04) #2B1(10;0.06) #3A/B2(8;0.03) #4B2(4;0.07) #5B2(2;0.04) #6B2(11;0.07) #7B2(10;0.10) #8C(13;0.14) #9A(21;0.04) #10B1(9;0.05) #11C(13;0.05) #12A(10;0.069) #13C(10;0.039) #14C(7;0.03) #15A(13;0.04) #16B2(15;0.06) #17A(12;0.07) #18B1(3;0.05) **89.7%**	#1A(11;0.07) **#2B1(2;0.15)** **#3A/B2(2;0.12)** #4B2(4;0.10) #5B2(0.03) #6B2(10;0.05) **#7B2(7;0.20)** **#8C(12;0.168)** #9A(20;0.11) **#10B1(11;0.09)** #11C(14;0.06) #12A(11;0.05) #13C(18;0.035) #14C(10;0.03) #15A(12;0.04) #16B2(11;0.04) **#17A(2;0.18)** #18B1(2;0.06) **92.9%**	#1A(15;0.07) #2B1(14;0.12) #3A/B2(14;0.06) #4B2(4;0.12) #5B2(5;0.21) #6B2(13;0.09) #7B2(7;0.08) #8C(9;0.167) **#9A(14;0.12)** #10B1(11;0.03) #11C(14;0.05) #12A(12;0.04) **#13C(13;0.043)** **#14C(12;0.05)** **#15A(13;0.05)** #16B2(13;0.09) #17A(7;0.13) **#18B1(7;0.18)** **91.8%**
